# Analyzing Histological Images Using Hybrid Techniques for Early Detection of Multi-Class Breast Cancer Based on Fusion Features of CNN and Handcrafted

**DOI:** 10.3390/diagnostics13101753

**Published:** 2023-05-17

**Authors:** Mohammed Al-Jabbar, Mohammed Alshahrani, Ebrahim Mohammed Senan, Ibrahim Abdulrab Ahmed

**Affiliations:** 1 Computer Department, Applied College, Najran University, Najran 66462, Saudi Arabia; iaalqubati@nu.edu.sa; 2 Department of Artificial Intelligence, Faculty of Computer Science and Information Technology, Alrazi University, Sana’a, Yemen; senan1710@gmail.com

**Keywords:** deep learning, ANN, fusion features, handcrafted, PCA, breast cancer, BreakHis

## Abstract

Breast cancer is the second most common type of cancer among women, and it can threaten women’s lives if it is not diagnosed early. There are many methods for detecting breast cancer, but they cannot distinguish between benign and malignant tumors. Therefore, a biopsy taken from the patient’s abnormal tissue is an effective way to distinguish between malignant and benign breast cancer tumors. There are many challenges facing pathologists and experts in diagnosing breast cancer, including the addition of some medical fluids of various colors, the direction of the sample, the small number of doctors and their differing opinions. Thus, artificial intelligence techniques solve these challenges and help clinicians resolve their diagnostic differences. In this study, three techniques, each with three systems, were developed to diagnose multi and binary classes of breast cancer datasets and distinguish between benign and malignant types with 40× and 400× factors. The first technique for diagnosing a breast cancer dataset is using an artificial neural network (ANN) with selected features from VGG-19 and ResNet-18. The second technique for diagnosing breast cancer dataset is by ANN with combined features for VGG-19 and ResNet-18 before and after principal component analysis (PCA). The third technique for analyzing breast cancer dataset is by ANN with hybrid features. The hybrid features are a hybrid between VGG-19 and handcrafted; and a hybrid between ResNet-18 and handcrafted. The handcrafted features are mixed features extracted using Fuzzy color histogram (FCH), local binary pattern (LBP), discrete wavelet transform (DWT) and gray level co-occurrence matrix (GLCM) methods. With the multi classes data set, ANN with the hybrid features of the VGG-19 and handcrafted reached a precision of 95.86%, an accuracy of 97.3%, sensitivity of 96.75%, AUC of 99.37%, and specificity of 99.81% with images at magnification factor 400×. Whereas with the binary classes data set, ANN with the hybrid features of the VGG-19 and handcrafted reached a precision of 99.74%, an accuracy of 99.7%, sensitivity of 100%, AUC of 99.85%, and specificity of 100% with images at a magnification factor 400×.

## 1. Introduction

Every organ in the human body comprises billions of cells that perform specific functions. Cancer is a group of abnormal cells that grow abnormally and without control and hinder the functioning of normal cells. Breast cancer is considered a life-threatening public health concern, as it is the second most common cancer in women among other types of cancer [1]. The number of breast cancer cases is increasing annually, according to the World Health Organization report. In 2020, more than 2.3 million women were diagnosed with breast cancer, and 685,000 women died of breast cancer worldwide [2]. Over the past five years, more than 7.8 million women have been diagnosed with breast cancer, which indicates its increased risk. Breast cancer arises in the breast’s glandular tissue, in the cells of the epithelium of the ducts 85% or the cells of the lobules 15%. The beginning of the growth of cancer cells does not show symptoms and spreads slowly in the ducts or the lobules. Over time, BC spreads and invades surrounding tissues (invasive breast cancer) and spreads to lymphocytes (regional metastasis). Breast cancer can be controlled if it is diagnosed early, and the patient undergoes appropriate treatment. There are many treatments for breast cancer, such as surgical removal, radiotherapy, chemotherapy, hormonal therapy, and targeted biological therapy. There are many techniques for detecting breast cancer such as radiography, MRI, CT, ultrasound, and tissue biopsies. Breast X-rays are a reference test for doctors to identify breast tumors and abnormal breast tissue changes. But these rays cannot distinguish between malignant and benign tumors. Therefore, a biopsy is necessary to take a sample of abnormal tissue and send it to laboratories for analysis [3]. Experts are working on adding hematoxylin and eosin (H&E) staining to the tissue sample to be analyzed, and it is considered as one of the obstacles that experts face in analyzing images [4]. There are many drawbacks due to factors such as sample orientation, laboratory protocols, operator variability, and variation of fluorophores for staining [5]. These obstacles are difficult for pathologists, who resort to magnifying the Whole Slide Images (WSI) sample with multiple magnification factors for accurate analysis. The tissue sample is analyzed under a microscope by expert pathologists, and the report concludes the presence of malignant or benign cancer cells. Although this is a reliable way to diagnose abnormal breast tissue effectively, there is some subjective issue with analyzing the same sample with different opinions among pathologists. There is also an acute shortage of experts of anatomical pathology, which leads to a delay in the sample analysis results for more than a month [6]. Thus, there is an urgent need for the development of automated techniques with the help of computers to overcome the limitations of manual diagnosis [7]. Artificial intelligence techniques, particularly in the field of machine learning, are being increasingly utilized to revolutionize the process of disease detection and diagnosis [8]. These techniques leverage the power of computational algorithms to analyze complex datasets, including medical images, patient records, genetic information, and other relevant clinical data. By training AI models on vast amounts of labeled data, these algorithms can learn to recognize patterns, anomalies, and potential disease indicators that may not be readily apparent to human observers [9]. This has the potential to greatly enhance the accuracy, speed, and efficiency of disease diagnosis, enabling early detection and intervention, and ultimately improving patient outcomes. Moreover, the continuous learning capability of AI systems allows for ongoing improvement and adaptation as new data become available, making them valuable tools in the field of medical diagnosis. Artificial intelligence techniques aim to automatically diagnose microscopic images of pathological tissues as benign or malignant and distinguish between their types. With the advent of WSI digital microscopes, slides can be scanned at various magnifications. The WSI with different magnification factors have opened the door for researchers to analyze microscopic pathological images using highly reliable artificial intelligence techniques. Deep learning techniques are considered the gold standard for medical image analysis because they have many layers to extract subtle features and also because of the similarity of biomarkers and biological characteristics between abnormal tissue samples of malignant and benign breast cancer types. Therefore, this study aimed to analyze the images of microscopic pathological tissues by hybrid methods and extract the features of the images by mixed methods between deep learning and handcrafted features [10].

The most important contributions to this study are as follows:

- Eliminating artifacts and increasing the contrast of edges of affected areas with histological images of the multi and binary classes of breast cancer data set for 40× and 400× magnification factors.
- Applying the PCA method after the VGG-19 and ResNet-18 models to eliminate redundant features and select the important features to reduce high dimensions.
- After reducing the feature dimensions of VGG-19 and ResNet-18, they have been integrated and fed to ANN for diagnosis.
- Incorporating the features of the VGG-19 and ResNet-18 models, then reducing the high dimensions and feeding them to the ANN for classification.
- Extraction of handcrafted features by integrating features of FCH, LBP, DWT and GLCM methods.
- Extracting hybrid features by integrating CNN features (VGG-19 and ResNet-18) with handcrafted features and feeding them to ANN for classification.
- Developing systems that can effectively assist pathologists and experts in diagnosing malignant and benign breast cancer tumors and discriminating between them.

The rest of the paper is organized as follows: Section 2 discusses relevant previous studies systems. Section 3 presents various methodologies and tools for diagnosing histological images of breast cancer and discrimination between its types. Section 4 summarizes the outcomes of the systems and their performance evaluation on multi and binary classes of breast cancer datasets. Section 5 reviews and compares the performance of systems. Section 6 concludes the study.

## 2. Related Work

Many previous studies aimed to discover the characteristics of abnormal tissues of the breast at an early stage and to distinguish between the types of breast cancer. The researchers devoted their efforts to reaching good results to differentiate between different types of breast cancer. This study was characterized by applying various hybrid methods, extracting features from many methods, then merging them to reach satisfactory results.

Sercan et al. [11] show that MITNET is trained on two breast cancer datasets. The network consists of MITNET-det and MITNET-rec to isolate cells and select points in WSIs. In MITNET-det, the deep features are extracted and combined by CSPDarknet. Then YOLOv4 is used to detect cells at different levels. The network reached a sensitivity of 82.9%, a precision of 58.6%, and an F1-score of 68.7%. Khuriwal et al. [12] proposed a deep learning network to classify the Wisconsin Breast Cancer dataset. Label Encoder and Standard Scaler have been applied for data optimization. About 11 of the 30 features were used for classification by deep and machine learning networks. Deep learning networks outperformed machine learning networks in performance. Wakili et al. [13] proposed the DenTnet’s method for classifying breast cancer images. The technique works based on transfer learning to extract features from the same distribution of the DenseNet model. The technique reached an accuracy of 97.85% and an AUC of 97% for classifying the two-class data set.

Kaplun et al. [14] used CNN to analyze breast cancer images and extract features from complex cancer cells. The results of testing are interpreted by local interpretations and quantifiable artificial intelligence. Man et al. [15] proposed the DenseNet121-AnoGAN model to correct the classification based on improved patches of pathological breast images and the discrimination of malignant and benign tissues. The model is evaluated on the BreaKHis dataset, which consists of two parts: first, the AnoGAN network to detect anomalies and examine the classified spots. Second, extract features from discriminatory spots by DenseNet. With images at a magnification factor of 40×, the model performed good accuracy for binary classification as either malignant or benign. Li et al. [16] proposed CNN to classify breast cancer and communicate feature information through DenseNet. The SENet module powers DenseNet to select essential features from malicious tissues. Gour et al. [17] developed the ResHist method for the histological diagnosis of breast cancer. The ResHist model learns rich features and classifies images as malignant or benign. Data augmentation, spot normalization, and affine transformation were applied. ResHist performed an accuracy of 84.34%; after using data augmentation, it performed an accuracy of 92.52%. Liew et al. [18] proposed the DLXGB network for classifying breast cancer histology images. Data augmentation and spot normalization have been applied to improve the images. Then DenseNet201 automatically learns feature extraction and is combined with a gradient boost classifier. The network classifies the image as benign or malignant. Sheikh et al. [19] designed a multi-scale and multi-feature model to determine features of texture and structure by integrating hierarchical features. The pattern predicts the presence of malignant or benign tissue. Lin et al. [20] proposed a CNN using a uniform experimental design to improve the parameters of CNN for diagnosing breast cancer histological images. With the optimization factor, the network reached an accuracy of 84.41%. Balaji et al. [21] proposed a social spider optimization (SSO) algorithm to improve CNN output to achieve the pathological classification of breast cancer biopsy images. The SSO algorithm adjusts the CNN network’s weights, achieving an accuracy of 95.91% and a sensitivity of 94.25%. Zahoor et al. [22] presented an optimization algorithm for diagnosing the histology of breast cancer cells. The algorithm combines both classifiers with and without parameters to create a high-efficiency binary classifier. The algorithm performed an accuracy of up to 80% for diagnosing breast cancer images. Hameed et al. [23] presented four pre-trained deep-learning models for diagnosing breast cancer images. All models were made through five-fold cross-validation processes. The models were fully trained and finely tuned, and all models’ average prediction was taken. The controlled VGG16 performed a competitive performance and reached an accuracy of 95.29%. Mewada et al. [24] proposed a CNN based on integrating the spectral features with the features of the CNN. Poor convergence is improved through convolutional layers, and then CNN training with integrating features. Boumaraf et al. [25] proposed machine learning and CNN algorithms to analyze breast cancer images. The machine learning algorithms were fed the advantages of three methods for classifying breast cancer images. CNN models train breast cancer images based on transfer learning. The accuracy of CNN was 76.77% for the eight-class classification and 94.05% for the two-class classification.

It is noted from previous studies that accuracy was the goal of every researcher. Therefore, this study focused on extracting features using hybrid methods to reach superior results in distinguishing between the types of breast cancer.

## 3. Materials and Methods

**Figure 1:**
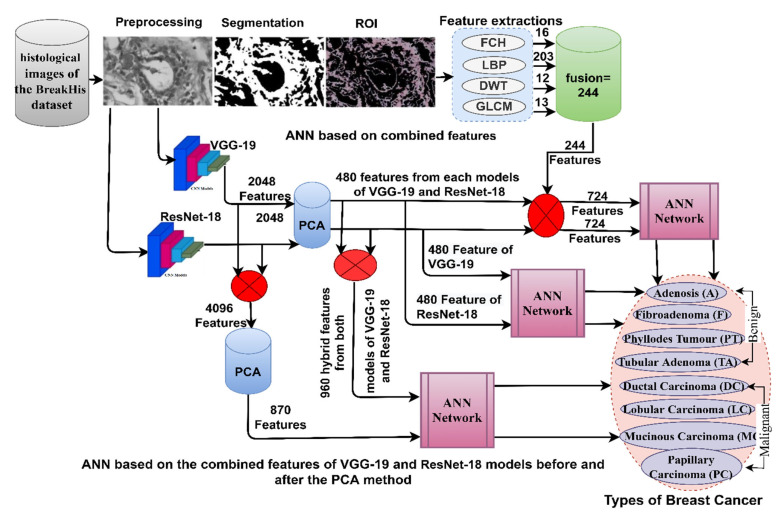
Methodology for diagnosing histological images of the breast cancer data set for multi and binary classes and distinguishing between malignant and benign types for 40× and 400× magnification factors.

This section discusses the methodologies applied to the histological diagnosis of breast cancer. Images have been improved for good results in the following stages as shown in Figure 1. The PCA has been used to clear redundant and unnecessary features and select the necessary features for the VGG-19 and ResNet-18. The first technique for histological diagnosis of breast cancer was by ANN with selected features of VGG-19 and ResNet-18 using PCA method. The third technique for diagnosing images of breast cancer was by ANN with the combined features of VGG-19 and ResNet-18 before and after dimension reduction. The third technique for diagnosing the images of breast cancer was by ANN with hybrid features of VGG-19 and ResNet-18 separately with handcrafted features.

### 3.1. Description of the BreakHis Dataset

**Figure 2:**
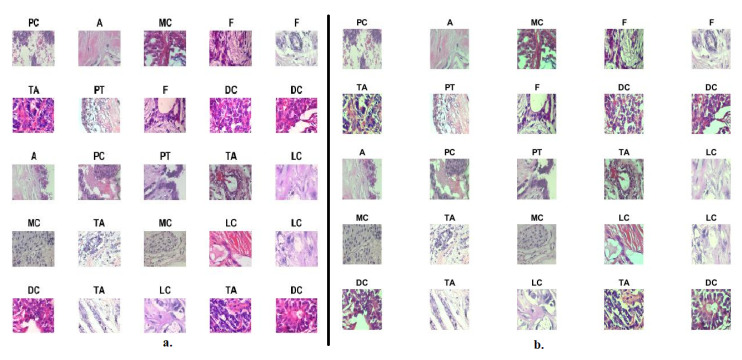
Images of abnormal breast tissue images with a magnification factor of 400×. (**a**) Before improving histology images of breast cancer. (**b**) After improving histology images of breast cancer.

Biopsies of the abnormal breast tissue of the patient are considered one of the essential techniques for the diagnosis of breast tissue. A biopsy is taken from the patient’s breast and sent to laboratories for analysis by pathologists. The biopsy is placed on glass slides and analyzed under a microscope. The biopsy is converted into WSI with different magnification factors to be presented to researchers and interested people to devote their efforts to its diagnosis using artificial intelligence. This study analyzed and categorized abnormal breast cancer tissue images in the BreakHis Dataset to evaluate systems. The data set consists of 7909 images of abnormal breast tissue collected in 2014 from the P&D laboratory in Brazil. The data set is divided into four classes (types) of tissues for malignant tumors and four classes (types) of tissues for benign tumors. The dataset contains histological images with magnification factors of 40×, 100×, 200×, and 400× [26]. This study aimed to classify histological images at 40× and 400× magnifications. The dataset contains 1995 histological images of 40× magnification factor distributed among eight classes as shown in Table 1: 114 images of class Adenosis (A), 253 images of class Fibroadenoma (F), 149 images of class Phyllodes Tumor (PT), 109 images of class Tubular Adenoma (TA) and 846 A picture of Ductal Carcinoma (DC), 156 images of Lobular Carcinoma (LC), 205 images of Ductal Carcinoma (MC), and 145 images of Papillary Carcinoma (PC). The dataset contains 1820 histological images with a 400× magnification factor distributed among eight classes as follows: 106 images of class A, 237 images of class F, 130 images of class PT, 115 images of class TA, 788 images of class DC, 137 images of class LC, 169 images of class MC and 138 images of the class PC. Figure 2a shows histological images of BreakHis data set.

### 3.2. Improving Breast Cancer Histological Images

This stage is to improve breast cancer tissue images and feed them to the next stages of biomedical image processing. Histological images are obtained from many different microscopes or scanners. Adding different H&E chemicals causes various colors to appear in the biopsy and is one of the obstacles to deep learning systems. Therefore, images must be improved before they are fed into deep-learning models. In this study, the images underwent several refinement processes to remove unwanted artifacts and increase the contrast of the affected tissues. Breast cancer tissue images have been improved by filter averaging to remove unwanted artifacts and the contrast limited adaptive histogram equalization (CLAHE) method to increase the contrast of affected tissues in regions of interest (ROI). The mean colors of histograms for the RGB channels were calculated. Moreover, scaling the colors of the texture images was adjusted to reach a consistent image of the colors [27].

All dataset images are subject to an average filter with a size of 5 × 5 to remove unwanted artifacts. The filter wraps with 25 pixels in the images each time; then selects a pixel to be processed and calculates the average of its 24 neighbors and then changes the value of the pixel which has been processed with the new average value as in Equation (1).

(1) $$Y\left(i\right)=\frac{1}{N}\overset{N-1}{\underset{a=0}{\sum }}S\left(i-a\right)$$

where $Y\left(i\right)$ indicates the input, $S\left(i-a\right)\;$ indicates the earlier input, and *N* indicates the number of image pixels.

To increase the contrast of the abnormal tissues of the breast cancer images, the images have been passed to the CLAHE method.

The CLAHE is an adaptation of the traditional CLAHE algorithm. CLAHE is a technique used to enhance the contrast of an image by equalizing the histogram of local image regions. The CLAHE modifies the original CLAHE algorithm to address some of its limitations and improve its performance. Here are the main steps of the CLAHE algorithm:

- Image Partitioning: The input image is divided into smaller blocks. The size of these blocks can vary depending on the application and image characteristics.
- Histogram Calculation: For each block, the histogram of pixel intensities is computed. The histogram represents the frequency distribution of pixel values within the block.
- Contrast Enhancement: The histogram of each block is then modified to enhance the contrast. The goal is to spread out the pixel intensities to cover a wider range of values, thus improving the visibility of details in both dark and bright regions.
- Contrast Limiting: To prevent over-amplification of noise or artifacts, a contrast limiting step is applied. This step sets a maximum limit on the amplification of pixel intensities within each block. It ensures that the enhancement process does not introduce unrealistic or exaggerated contrast changes.
- Histogram Equalization: After the contrast enhancement and limiting steps, the modified histogram is used to redistribute the pixel values within each block. This histogram equalization step ensures that the pixel intensities are spread out evenly across the available dynamic range.
- Block Reconstruction: Finally, the enhanced blocks are combined back to reconstruct the output image. Depending on the implementation, blending or overlap techniques can be used to smooth the transitions between blocks and create a visually coherent result.

The CLAHE algorithm improves upon the traditional CLAHE method by incorporating contrast limiting and modifying the histogram equalization process. These modifications help in achieving a more balanced enhancement, avoiding excessive amplification of noise, and maintaining a natural appearance in the resulting image. The CLAHE is commonly used in various image processing applications where contrast enhancement is desired, such as medical imaging, satellite imagery, and computer vision tasks. The method continues until each pixel has been treated with the histological image. Finally, after processing each pixel of the image is completed, the resulting image is enhanced with clear contrast. Figure 2b set of histological images of improved data set.

### 3.3. ANN According to Deep Learning Features

The department offers hybrid technology based on ANN and deep learning models. Pre-trained CNN models do not reach good results for diagnosing breast cancer histology and distinguishing between types of tumors. Moreover, training and evaluating the data set requires an expensive and time-consuming computer [28]. So, this technique is to resolve these updates [29]. Deep learning models extract features from images of breast cancer tissue, select the most significant attributes for each type of tumor and reduce the dimensions by PCA. The important features are sent to the ANN to split them during the training and testing phase, then train them, adjust the weights, and test their performance on the new features (testing).

#### 3.3.1. Features of Deep Learning

What distinguishes deep learning is that they have many layers that have the ability to extract deep features from each disease and distinguish it from the other. The input layers for VGG-19 and ResNet-18 models were fed with improved histological images of the breast cancer data set containing four malignant breast cancer types and four benign breast cancer types [30]. Each layer works with a specific task, and at a high level, each layer has millions of neurons connected to the same layer and other layers by weights. Each layer produces a specific task through precise arithmetic operations [31]. In this work, the main task of the VGG-19 and ResNet-18 models is to extract features from the first convolutional layer to the last convolutional layer and reduce neurons through pooling layers and pass through auxiliary layers to perform a particular task [32].

The CNNs are designed to automatically extract features from input data, especially in computer vision tasks such as image classification. By stacking multiple convolutional, pooling, and fully connected layers, CNNs learn hierarchical representations of the input data. The initial layers capture local and low-level features, while subsequent layers learn more abstract and high-level representations. This hierarchical feature extraction allows CNNs to automatically learn and recognize complex patterns and structures within the input data, making them highly effective for various computer vision tasks.

The VGG-19 and ResNet-18 models are popular deep learning architectures that are commonly used in computer vision tasks. Both VGG-19 and ResNet-18 have their strengths and are suitable for different use cases. VGG-19 is known for its accuracy and is often used in scenarios where high performance is the priority, even at the expense of computational cost. On the other hand, ResNet-18 provides a good trade-off between accuracy and efficiency, making it suitable for applications where computational resources are constrained or real-time processing is required.

The workflow in convolutional neural network (CNN) models typically involves the following steps: [33].

- Data Preprocessing: The input data, usually images, undergoes preprocessing steps to ensure uniformity and compatibility with the CNN model. This may involve resizing the images to a consistent size, normalizing pixel values, and applying any required transformations such as rotation, cropping, or augmentation.
- Model Architecture Definition: The CNN model’s architecture is defined, specifying the arrangement and configuration of layers. This includes determining the number and types of layers (convolutional, pooling, and fully connected), their sizes, and any additional components such as dropout or batch normalization layers [34].
- Model Compilation: Once the architecture is defined, the model is compiled by specifying the loss function, optimizer, and optional metrics. The loss function measures the model’s performance during training, the optimizer updates the model’s weights based on the computed loss, and the metrics evaluate the model’s performance during training and evaluation [35].
- Training: The CNN model is trained on a labeled dataset. During training, input data is fed into the model, and the model’s weights are adjusted iteratively to minimize the loss function. This involves forward propagation, where the input data passes through the layers, and backward propagation (backpropagation), where gradients are computed and used to update the weights via the optimizer.
- Validation: Periodically, during training, a validation set is used to evaluate the model’s performance on unseen data. This helps monitor the model’s progress, detect overfitting, and make decisions regarding hyperparameter tuning or early stopping.

It is important to note that CNN workflows can vary depending on the specific task, dataset, and requirements. Different techniques such as transfer learning, regularization, or hyperparameter tuning may also be incorporated into the workflow to improve model performance.

The final layer of VGG-19 and ResNet-18 models produces high-dimensional features and is preserved in vectors of size 1995 × 2048 and 1820 × 2048 for the breast cancer data set for the magnification factors 40× and 400×, respectively. These vectors were fed to the PCA method to select features and reduce dimensions. The PCA produced vectors with a size of 1995 × 480 and 1820 × 480 for the breast cancer data set for the magnification factors 40× and 400×, respectively.

#### 3.3.2. PCA Algorithm

The principal component analysis (PCA) algorithm is a dimensionality reduction technique that aims to find a lower-dimensional representation of a dataset while preserving its most important information [36]. The underlying principle of PCA is to identify a new set of orthogonal axes, called principal components, along which the data has the highest variance. The steps involved in the PCA algorithm are as follows [37]:

1. Standardize the data: PCA requires that the input data are standardized. This step ensures that all features contribute equally to the analysis.
2. Compute the covariance matrix: The covariance matrix is calculated based on the standardized data. It provides information about the relationships between the different features and their variances.
3. Compute the eigenvectors and eigenvalues: They are obtained from the covariance matrix. The eigenvectors represent the principal components, and the corresponding eigenvalues measure the amount of variance explained by each principal component. The eigenvectors are orthogonal to each other.
4. Select the principal components: The principal components are chosen based on the eigenvalues. The components associated with higher eigenvalues capture more variance in the data and are considered more important.
5. Project the data onto the new feature space: The original data are transformed into the new feature space spanned by the selected principal components. This is done by computing the dot product between the standardized data and the eigenvectors.

The main principle behind PCA is to reduce the dimensionality of the data by projecting it onto a lower-dimensional subspace while retaining the maximum amount of information. By selecting a subset of the principal components that capture most of the variance, PCA allows for a compact representation of the data while minimizing the loss of information.

PCA has several applications, including data visualization, noise reduction, feature extraction, and data compression. It is particularly useful when dealing with high-dimensional data or when looking for patterns or relationships in the data that might not be easily apparent in the original feature space.

PCA is commonly used for feature reduction in several scenarios:

High-dimensional data: When dealing with datasets that have a large number of features, PCA can effectively reduce the dimensionality of the data. This is particularly useful when the high dimensionality poses challenges in terms of computational complexity, memory requirements, or model overfitting. By selecting a subset of principal components, PCA allows for a more compact representation of the data without losing significant information.

#### 3.3.3. The Classification Using ANN

**Figure 3:**
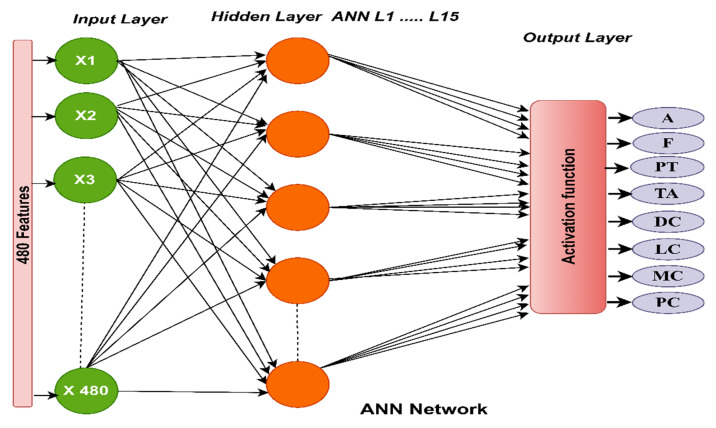
ANN framework for receiving and diagnosing images of breast cancer tissue and distinguishing between them.

In this study, the ANN receives the selected low-dimensional features of VGG-19 and ResNet-18 models. ANN is characterized by its high performance in diagnosing breast cancer histology [38]. The ANN consists of three basic layers that receive the features and process them to perform the task of diagnosing each inputted image. In this study, the ANN input layer receives features of 1995 × 480 and 1820 × 480 for the magnification factors 40× and 400×, respectively. Thus, the input layer contains 480 features of both feature vectors 1955 × 48 and 1820 × 480 as shown in Figure 3. The features pass to the hidden layers, which consist of 15 hidden layers connected by tuned weights. All tasks are done in hidden layers, and each time the weights are updated, the minimum square error (*MSE*) is calculated [39]. The network continues to train the data (features) and change the weights in each iteration until it reaches the *MSE* between the expected $y_{i}$ and actual $z_{i}$ values, as in Equation (2) [40]. Each feature vector is sorted into a specific class. The output layer contains eight neurons, where each neuron represents one class in the data set. The multiclass dataset contains eight classes, so the output layer has eight neurons.

(2) $$\;MSE=\frac{1}{m}\overset{m}{\underset{i=1}{\sum }}\;{\left(\;y_{i}-z_{i}\right)}^{2}$$

where *m* is the number of data features, $y_{i}$ is the expected value, and $z_{i}$ is the actual value.

**Figure 4:**
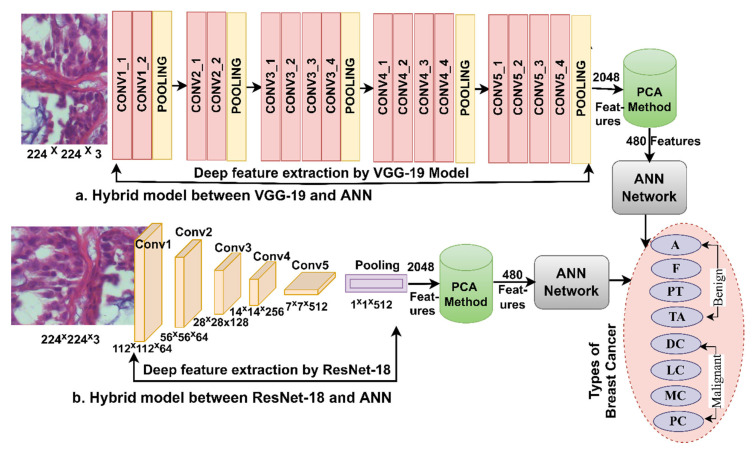
Hybrid methodology for diagnosis of histological images of breast cancer and distinguishing between them.

Figure 4 describes the methodology for diagnosing breast cancer tissue images and distinguishing their types using a hybrid technique between deep learning and ANN models.

### 3.4. ANN according to the Merge of Deep Learning Features

**Figure 5:**
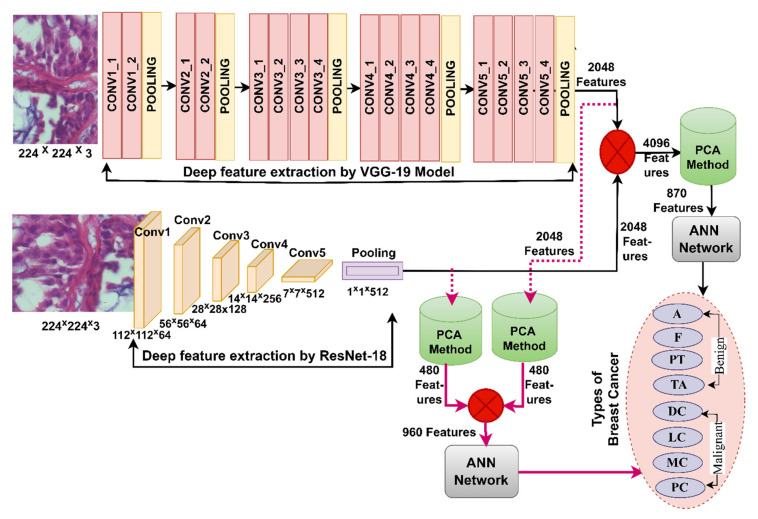
A hybrid methodology for diagnosing and distinguishing histological images of breast cancer based on integrating deep learning features.

This section presents a hybrid methodology for diagnosing images of the breast cancer dataset and distinguishing between its benign and malignant types. Some biomedical datasets, including the BreakHis dataset, do not yield satisfactory results when classified by pre-trained deep learning models [41]. Therefore, this technique solves these challenges and achieves satisfactory results for diagnosing eight benign and malignant breast cancer types. The technology integrates the features of both VGG-19 and ResNet-18 models and classifies them by ANN, as shown in Figure 5.

The hybrid methodology consists of two systems for diagnosis of images and discrimination of breast cancer types. In both systems, the improved histological images of breast cancer pass through many stages. The first system is as follows: First, filters are applied to optimize the histological images of breast cancer and fed separately to VGG-19 and ResNet-18. Second, VGG-19 and ResNet-18 pass images to many layers for analysis, extract deep features from all images and save them in 1995 × 2048 and 1820 × 2048 vectors for 40× and 400× magnification factors, respectively for both VGG-19 and ResNet-18 separately. Third, the features of both models VGG-19 and ResNet-18 are merged into vectors size 1995 × 4096 and 1820 × 4096 for magnification factors 40× and 400×, respectively. Fourth, the repetitive, unimportant features are deleted and the high dimensions are reduced by PCA method and saved in vectors of size 1995 × 870 and 1820 × 870 for magnification factors 40× and 400×, respectively. Fifth, the classification stage uses the ANN network, which receives vectors of size 1995 × 870 and 1820 × 870 for magnification factors 40× and 400×, respectively. Finally, the ANN was tuned to segment the dataset, train it, and test its performance.

For the second system, the first and second stages are same as in the first system. Third, the repetitive and unimportant features were removed and thus the high dimensions of the VGG-19 model were reduced by PCA method and saved in vectors of size 1995 × 480 and 1820 × 480 for both magnification factors 40× and 400×, respectively. Fourth, the repetitive and unimportant features were removed and thus reduced the high dimensions of the ResNet-18 model by PCA method and saved in vectors of size 1995 × 480 and 1820 × 480 for both magnification factors 40× and 400×, respectively. Fifth, the low-dimensional features of the VGG-19 and ResNet-18 models are combined and saved in vectors of size 1995 × 960 and 1820 × 960 for both magnification factors 40× and 400×. The features of the two CNN models are merged in a serial manner, where the feature vectors of the VGG16 model are combined with the feature vectors of the ResNet18 model in a serial manner using a function.

Sixth, the classification stage uses the ANN network, which receives vectors of size 1995 × 960 and 1820 × 960 for magnification factors 40× and 400×, respectively. Finally, the ANN was tuned to segment the dataset, train it, and test the new sample’s performance.

### 3.5. ANN according to the Hybrid Features

**Figure 6:**
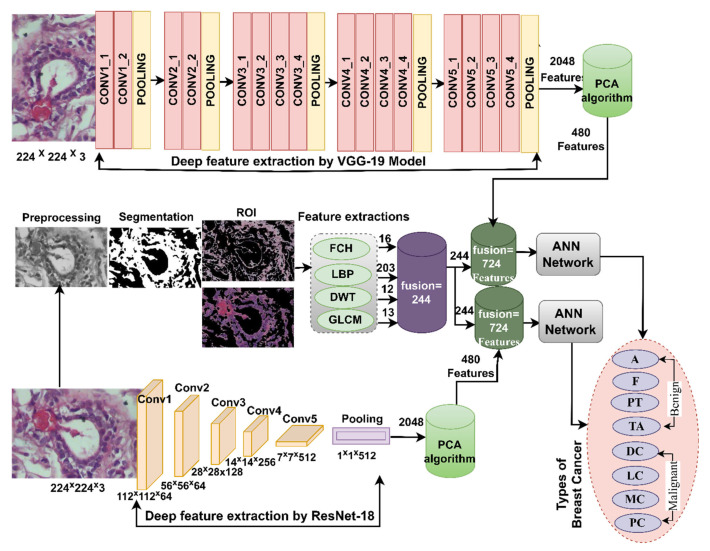
A hybrid methodology for diagnosing and distinguishing breast cancer histological images based on integrating features of deep learning and handcrafted features.

This section presents a novel hybrid methodology for the diagnosis of breast cancer from histological images and for distinguishing between its benign and malignant types. The technology extracts the features of the VGG-19 and ResNet-18 models separately and combines them with the handcrafted features extracted by FCH, LBP, DWT, and GLCM methods and categorized by ANN, as shown in Figure 6. Hence, the combined features of this method are called hybrid features.

In the field of machine learning and artificial intelligence, handcrafted features were commonly used before the advent of deep learning and the rise of end-to-end learning approaches. These features are designed to capture relevant information and encode it in a format that is suitable for machine learning algorithms. Handcrafted features can be derived from various types of data, such as images, text, audio, or any other form of structured or unstructured data. For example, in image processing, handcrafted features may include color histograms, texture descriptors, edge detectors, or other manually engineered representations that highlight specific aspects of an image. The process of creating hand-crafted features involves careful analysis of the data, domain knowledge, and iterative experimentation. Experts identify relevant attributes or properties that are likely to be informative for the task at hand and design algorithms or procedures to extract and quantify those features from the raw data. In this study, color, geometric, and texture features were extracted by FCH, LBP, DWT, and GLCM algorithms.

The hybrid methodology consists of two systems for diagnosis of histological images and discrimination of breast cancer types. In both systems, the improved histological images of breast cancer pass through many stages. First, filters have been applied to improve histological images of breast cancer and fed separately to VGG-19 and ResNet-18. Second, VGG-19 and ResNet-18 pass images to many layers for analysis, extracting deep features from all images and saving them in vectors sizes of 1995 × 2048 and 1820 × 2048 for magnification factors of 40× and 400×, respectively for VGG-19 and ResNet-18 separately. Third, repetitive and unimportant features are deleted, and high dimensionality is reduced by the PCA method and saved in vectors with sizes of 1995 × 480 and 1820 × 480 for 40× and 400× magnification factors, respectively. Fourth, extraction of handcrafted features by passing improved histological images of breast cancer to traditional methods FCH, LBP, DWT, and GLCM. All conventional methods produce 244 features distributed as follows.

The ROI for histological images improved of breast cancer is passed to the FCH color algorithm to extract color features according to the method of fuzzy logic. This algorithm is essential for distinguishing malignant and benign breast cancers through biopsy colors. The algorithm creates several bin histograms according to the colors of the histogram [42]. The algorithm begins by assigning a bin histogram to each color, noting the distribution according to fuzzy logic. The colors in all bin histograms are all colors of the histological image. Any two colors in one bin histogram are the same even if they differ. Thus, 16 features are extracted from each image and preserved in vectors of size 1995 × 16 and 1820 × 16 for magnification factors 40× and 400×, respectively.

The ROI for histological images improved of the breast cancer data set is passed to the LBP binary texture algorithm to extract the binary texture features according to the gray matrix. This algorithm is easy and important to distinguish between benign and malignant breast cancers by the texture of the biopsy [43]. The algorithm is set to 3 × 3, which indicates that the LBP extracts spatial data in each iteration of nine pixels (one targeted pixel and eight adjacent pixels). In each iteration, the $g_{c}$ pixel is targeted and changed according to the values of eight neighbouring pixels $g_{p}$ based on Equation (3). The algorithm produces 203 textures for each image and saves them in vectors of size 1995 × 203 and 1820 × 203 for magnification factors 40× and 400×, respectively.

(3) $$LBP_{R,P}=\overset{P-1}{\underset{i=0}{\sum }}s\left(g_{p}-g_{c}\right)2^{i}$$

where $g_{c}$ refers to the processed pixel, $g_{p}$ refers to the closest pixels R refers to the radius, and P refers to the number of closest pixels.

The ROI for histological images improved of the breast cancer data set is passed to the DWT algorithm to extract geometric features. This algorithm divides the image into four regions and assigns a filter to each region. The first region (upper left) is passed to a low filter to analyze the first region of the histological image based on approximate parameters and extract three statistical features. The second region (upper right) and the third region (lower left) are passed to the Low-High and High-Low filters, respectively, to analyze the second and third regions of the histological image based on the detailed parameters and extract six features statistical. The fourth region (lower right) is passed to a high filter to analyze the fourth segment of the histological image based on detailed parameters and extract three statistical features. The method produces 12 geometric features for each image and saves them in vectors sizes of 1995 × 12 and 1820 × 12 for 40× and 400× magnification factors, respectively.

The ROI for the histological images improved of the breast cancer data set is passed to the GLCM texture algorithm to extract the coarse and smooth texture features from the grayscale matrix. The algorithm extracts spatial data based on the central pixel and its neighbors. The target pixel is compared with the neighboring pixels, and the algorithm selects the adjacent pixels according to the angles around the target pixel, adjacent pixels’ angles neighbors are 0°, 45°, 90°, 135° with the d distance measure [44]. The algorithm selects coarse regions, which have sharply different pixels, and smooth regions, which have equal pixels. The method produces 13 distinct textures for each image and saves them in vectors of 1995 × 16 and 1820 × 16 for magnification factors 40× and 400×, respectively.

Fifth, obtaining handcrafted features by integrating the features of the four traditional methods into hybrid vectors with sizes of 1995 × 244 and 1820 × 244 for magnification factors 40× and 400×, respectively.

Sixth, obtaining the radiological features by incorporating the features of the VGG-19 model with handcrafted for obtaining hybrid vectors features of the size 1995 × 724 and 1820 × 724 for magnification factors of 40× and 400×, respectively.

Seventh, obtaining the radiographic features by integrating the features of the ResNet-18 model with handcrafted features for obtaining hybrid vectors features of the size 1995 × 724 and 1820 × 724 for magnification factors of 40× and 400×, respectively.

Eighth, feeding the hybrid features to the ANN which splits the data set to train the network and evaluate its performance while testing new samples.

## 4. Evaluate the Systems

### 4.1. Split of Breast Cancer Data Set (BreakHis)

In this study, images of breast cancer tissues were analyzed using many different systems to diagnose and differentiate between benign and malignant tumors. The data set contains 1995 and 1820 images with magnification factors of 40× and 400× for eight types of tumors divided into four types of benign and four types of malignant. Benign tumors contain 625 and 588 histological images of magnification factors 40× and 400×, divided among the four classes of benign tumors unbalanced, as shown in Table 1. Malignant tumors contain 1370 and 1232 histological images of magnification factors 40× and 400× divided among the four classes of malignant tumors unbalanced, as shown in Table 1. Thus, the data set consists of eight unbalanced classes. All systems split the data set into 80% during training and validation to adjust parameters and 20% as new samples to test the ability of the systems to diagnose new samples. All the proposed technologies were implemented by the MATLAB 2018b operational environment on a 10th generation i5 processor with 16 GB RAM and 4 GB GPU.

### 4.2. Systems Evaluation Metrics

All systems produced a confusion matrix as the output of their performance on the breast cancer dataset. The confusion matrix contains all systems performance test samples that are correctly classified (TP and TN) and incorrectly (FP and FN) [45]. The systems were evaluated through scales as with Equations (4)–(8).

(4) $$\mathrm{Precision}=\frac{\mathrm{TP}}{\mathrm{TP}+\mathrm{FP}}\;*\;100\%\;$$

(5) $$\mathrm{Accuracy}=\frac{\mathrm{TN}+\mathrm{TP}}{\mathrm{TN}+\mathrm{TP}+\mathrm{FN}+\mathrm{FP}}\;*\;100\%$$

(6) $$\mathrm{Sensitivity}=\frac{\mathrm{TP}}{\mathrm{TP}+\mathrm{FN}}\;*\;100\%$$

(7) $$\mathrm{AUC}\;=\frac{\mathrm{TP}\;\mathrm{Rate}}{\mathrm{FP}\;\mathrm{Rate}}$$

(8) $$\mathrm{Specificity}=\frac{\mathrm{TN}}{\mathrm{TN}+\mathrm{FP}}\;*\;100$$

### 4.3. Balance Dataset Classes and Overcome the Overfitting Problem

For effective performance by CNN models, a vast data set is needed to train the systems with a sufficient number of images, which is not available in most biomedical data sets. Because of the lack of dataset, images cause overfitting. Moreover, the accuracy of the CNN models tends to the classes that have more images, and therefore the performance of the CNNs tends to the classes of the majority. Therefore, these two challenges represent obstacles to CNN models, which were overcome by applying data augmentation technology.

Data augmentation helps improve model performance and generalization by introducing more diversity and variability into the training data. Here is how data augmentation typically works:

Image Transformations: Apply a variety of image transformations to the original dataset. Some common transformations include:

Rotation: Rotate the image by a certain angle.
Flip: Flip the image horizontally or vertically.
Translation: Shift the image in the *x* and *y* directions.
Scaling: Resize the image to a different size.
Shearing: Distort the image by shifting one side along a certain axis.
Zooming: Zoom in or out on the image.
Randomization: Apply the image transformations randomly, with different parameters for each transformation. This randomization introduces diversity and prevents the model from relying too heavily on specific patterns or orientations.

This technology artificially increases the images of the data set through many operations such as rotation, shifting, flipping, rotating the image in several angles, and others. Thus, the problem of overfitting was solved by generating many images in the training phase [46]. When increasing the data set images, the unbalanced data set was considered, and the images were increased unevenly to make the data set balanced. The images of minority classes were more than those of the majority classes. Thus, the problem of an unbalanced data set was overcome [47]. Table 2 shows the distribution of histological images of benign and malignant breast cancer before applying the data augmentation method and comparing it with the number of images after the data augmentation method. It is noted that the breast cancer data set for the magnification factors of 40× and 400× became balanced after applying the data augmentation. It was also noticed that the number of images increased differently from one class to another, which balanced the data set.

### 4.4. Results of ANN According to Deep Learning Features

The section summarizes the results obtained by ANN when fed with selected low-dimensional features extracted by VGG-19 and ResNet-18 models to analyze histological images for early diagnosis and discrimination between benign and malignant breast cancer types. VGG-19 and ResNet-18 models produce high dimensional features, so redundant features were omitted and essential features were selected for dimensionality reduction by PCA, and features were saved in vectors for both magnification factors of 40× and 400× for VGG-19 and ResNet-18 separately. The ANN receives the selected features and splits them up to train and validate the system and test the system on new samples.

**Figure 7:**
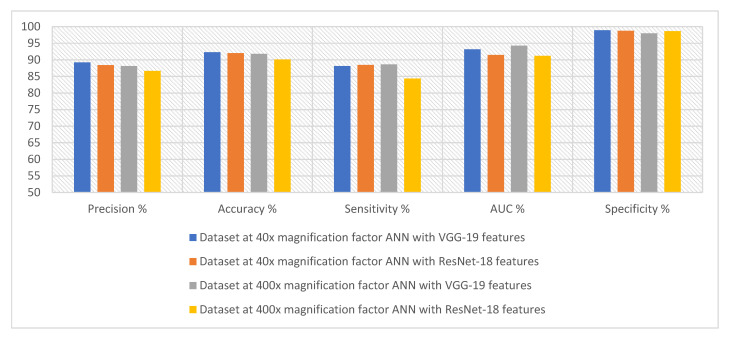
ANN performance with selected features and low-dimensionality of VGG-19 and ResNet-18.

Table 3 and Figure 7 summarize the performance of the ANN with selected low-dimensional features from the VGG-19 and ResNet-18 models of the breast cancer data set for all benign and malignant classes (multi-classes) of the magnification factors 40× and 400×. When fed to an ANN with VGG-19 features, it slightly outperforms compared to when fed ResNet-18 features.

First, when diagnosing histological images of breast cancer with a magnification factor of 40×, with the features of VGG-19, ANN reached a precision of 89.25%, an accuracy of 92.3%, sensitivity of 88.12%, AUC of 93.15%, and specificity of 98.88%. Whereas, with the features of ResNet-18, ANN achieved a precision of 88.38%, an accuracy of 92%, a sensitivity of 88.45%, an AUC of 91.48%, and a specificity of 98.75%.

Second, when diagnosing histological images of breast cancer with a magnification factor of 400×, with the features of VGG-19, ANN reached a precision of 88.13%, an accuracy of 91.8%, sensitivity of 88.62%, AUC of 94.25%, and specificity of 97.85%. Whereas, with the features of ResNet-18, ANN achieved a precision of 86.63%, an accuracy of 90.1%, a sensitivity of 84.38%, an AUC of 91.21%, and a specificity of 98.63%.

Table 4 summarizes the performance of the ANN with selected low-dimensional features of the VGG-19 and ResNet-18 models for the breast cancer data set as benign and malignant (binary classification) for 40× and 400× magnification factors. When fed to an ANN with VGG-19 features, it slightly outperforms compared to when fed it with ResNet-18 features.

First, when diagnosing histological images of breast cancer with a magnification factor of 40×, ANN with the features of VGG-19 reached a precision of 93.5%, an accuracy of 95%, sensitivity of 95.17%, AUC of 96.34%, and specificity of 95.58%. Whereas, with the features of ResNet-18, ANN achieved a precision of 93.28%, an accuracy of 94.2 a sensitivity of 94.66%, an AUC of 95.87%, and a specificity of 95.41%.

Second, when diagnosing histological images of breast cancer with a magnification factor of 400×, ANN with the features of VGG-19 reached a precision of 94.31%, an accuracy of 95.1%, sensitivity of 95.72%, AUC of 96.78%, and specificity of 96.35%. Whereas, with the features of ResNet-18, ANN achieved a precision of 92.5%, an accuracy of 94%, a sensitivity of 94.43%, an AUC of 93.82%, and a specificity of 95.73%.

**Figure 8:**
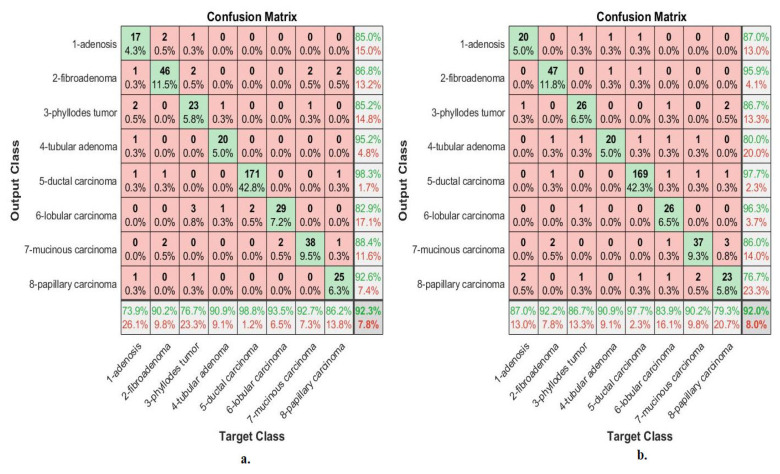
Confusion matrix for the multi classes breast cancer dataset with 40× magnification factor from ANN with features selected from (**a**) VGG-19 (**b**) ResNet-18.

Figure 8 describes the confusion matrix of performance of an ANN with selected features from the VGG-19 and ResNet-18 models for multi-classes diagnosis of histological images of breast cancer with a magnification factor of 40× and the distinction between benign and malignant types. ANN, when fed with selected features low-dimensional of VGG-19, it achieves a diagnostic accuracy for all classes: for Adenosis of 73.9%, for Fibroadenoma of 90.2%, for Phyllodes Tumor of 76.7%, for Tubular Adenoma of 90.9%, for Ductal Carcinoma of 98.8%, for Lobular Carcinoma of 93.5%, for Mucinous Carcinoma of 92.7%, and the Papillary Carcinoma of 86.2%. While with the features of ResNet-18, ANN achieved accuracy for all classes: for Adenosis of 87%, for Fibroadenoma of 92.2%, for Phyllodes Tumor of 86.7%, for Tubular Adenoma of 90.9%, for Ductal Carcinoma of 97.7%, for Lobular Carcinoma of 83.9%, for Mucinous Carcinoma of 90.2%, and the Papillary Carcinoma of 79.3%.

**Figure 9:**
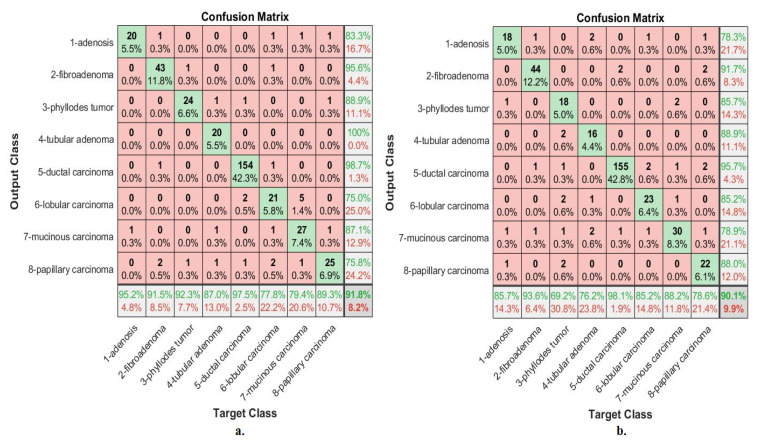
Confusion matrix for the multi classes breast cancer dataset with 400× magnification factor from ANN with features selected from (**a**) VGG-19 (**b**) ResNet-18.

Figure 9 describes the confusion matrix of performance of an ANN with selected features from the VGG-19 and ResNet-18 models for multi-classes diagnosis of histological images of breast cancer with a magnification factor of 400× and the distinction between benign and malignant types. ANN, when fed with selected low-dimensional features of VGG-19, it achieves a diagnostic accuracy for all classes: for Adenosis of 95.2%, for Fibroadenoma of 91.5%, for Phyllodes Tumor of 92.3%, for Tubular Adenoma of 87%, for Ductal Carcinoma of 97.5%, for Lobular Carcinoma of 77.8%, for Mucinous Carcinoma of 79.4%, and the Papillary Carcinoma of 89.3%. While with the features of ResNet-18, ANN achieved accuracy for all classes: for Adenosis of 85.7%, for Fibroadenoma of 93.6%, for Phyllodes Tumor of 69.2%, for Tubular Adenoma of 76.2%, for Ductal Carcinoma of 98.1%, for Lobular Carcinoma of 85.2%, for Mucinous Carcinoma of 88.2%, and the Papillary Carcinoma of 78.6%.

**Figure 10:**
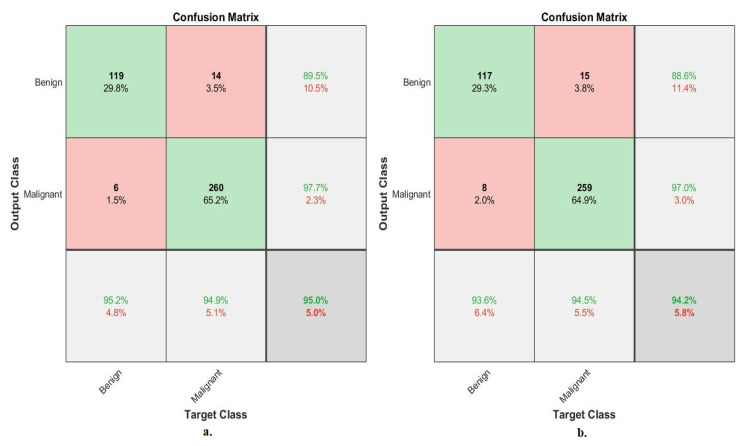
Confusion matrix for the binary classes breast cancer dataset with 40× magnification factor from ANN with features selected from (**a**) VGG-19 (**b**) ResNet-18.

Figure 10 describes the confusion matrix of the performance of an ANN with selected features from the VGG-19 and ResNet-18 models for binary classes diagnosis of histological images of breast cancer with a magnification factor of 40× for the distinction between benign and malignant types. ANN, when fed with selected low-dimensional features of VGG-19, it achieves a diagnostic accuracy for all classes: for the benign class of 95.2%, for the malignant class of 94.9%. While with the features of ResNet-18, ANN achieved accuracy for all classes: for benign of 93.6%, for malignant of 94.5%.

**Figure 11:**
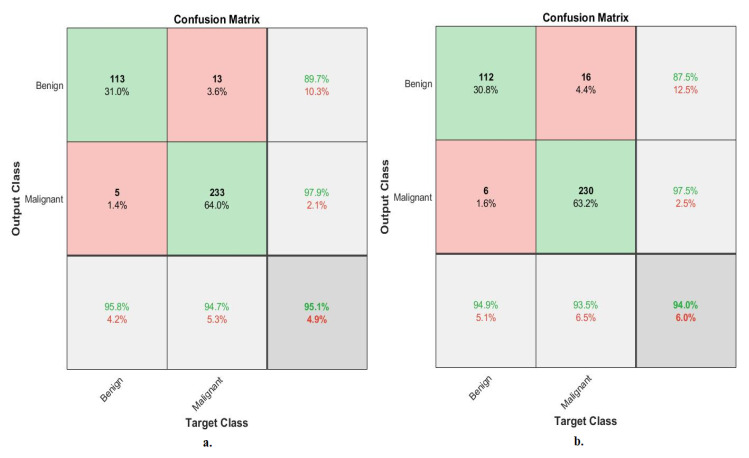
Confusion matrix for the binary classes breast cancer dataset with 400× magnification factor from ANN with features selected from (**a**) VGG-19 (**b**) ResNet-18.

Figure 11 describes the confusion matrix of the performance of an ANN with selected features from the VGG-19 and ResNet-18 models for binary classes and diagnosis of histological images of breast cancer with a magnification factor of 400× for the distinction between benign and malignant types. ANN, when fed with selected features low-dimensional of VGG-19, it achieves a diagnostic accuracy for all classes: for benign of 95.8%, for malignant of 94.7%. While with the features of ResNet-18, ANN achieved an accuracy for all classes: for benign of 94.9%, for malignant of 93.5%.

### 4.5. Results of ANN According to the Merge of Deep Learning Features

The section summarizes the results obtained by ANN when fed with low-dimensional hybrid features from VGG-19 and ResNet-18 models to analyze histological images for early diagnosis and discrimination of benign and malignant breast cancer types. The idea of this approach is as follows: First, extract features from VGG-19 and ResNet-18, then combining the features of the two models into the feature vectors. The feature vectors are fed to PCA to delete redundant features and select important features to reduce high dimensionality. Second, extract features from VGG-19 and ResNet-18, then deleting redundant features and select the important features to reduce high dimensionality separately for each model. The features selected are combined from the two low-dimensional models. The ANN receives features vectors and splits them to train and validate the system and test the system on new samples.

**Figure 12:**
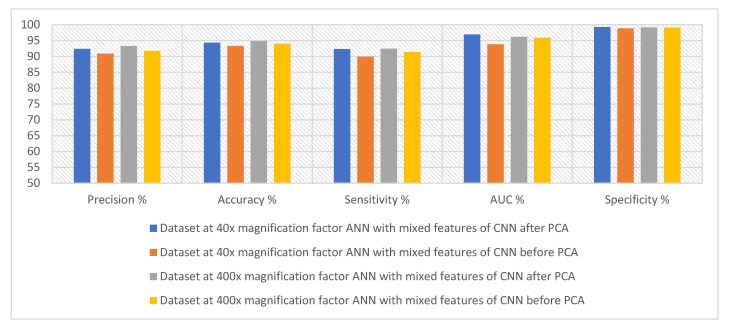
ANN performance with mixed features of VGG-19 and ResNet-18.

Table 5 and Figure 12 summarize the performance of the ANN with selected hybrid features of the VGG-19 and ResNet-18 models of the breast cancer data set for all benign and malignant classes (multi classes) at 40× and 400× magnification factors. It is worth noting that the methodology has two systems first, merging the high-dimensional features (before reducing the dimensions), then deleting the recurring features and selecting the important features to reduce the dimensions. Second, the merging of low-dimensional features, which means first reducing the dimensions of each model and then merging them. The performance of ANN with combined features after dimension reduction outperforms the performance of it with combined features before dimension reduction.

First, when diagnosing breast cancer histological images with a magnification factor of 40×, the ANN with mixed features after dimensionality reduction for VGG-19 and ResNet-18 reached a precision of 92.38%, an accuracy of 94.3%, sensitivity of 92.29%, AUC of 96.88%, and specificity of 99.25%. While the ANN with mixed features before dimensionality reduction for VGG-19 and ResNet-18 reached a precision of 90.88%, an accuracy of 93.3%, sensitivity of 89.86%, AUC of 93.79%, and specificity of 98.86%.

Second, when diagnosing breast cancer histological images with a magnification factor of 400×, the ANN with mixed features after dimensionality reduction for VGG-19 and ResNet-18 reached a precision of 93.23%, an accuracy of 94.8%, sensitivity of 92.41%, AUC of 96.17%, and specificity of 99.13%. While the ANN with mixed features before dimensionality reduction for VGG-19 and ResNet-18 reached a precision of 91.75%, an accuracy of 94%, sensitivity of 91.38%, AUC of 95.89%, and specificity of 99.1%.

Table 6 summarizes the performance of the ANN with selected hybrid features of the VGG-19 and ResNet-18 models of the breast cancer data set for benign and malignant classes (binary classes) at 40× and 400× magnification factors.

First, when diagnosing breast cancer histological images with a magnification factor of 40×, the ANN with mixed features after dimensionality reduction for VGG-19 and ResNet-18 reached a precision of 97.34%, an accuracy of 97.5%, sensitivity of 97.82%, AUC of 98.86%, and specificity of 97.91%. While the ANN with mixed features before dimensionality reduction for VGG-19 and ResNet-18 reached a precision of 96.12%, an accuracy of 97%, sensitivity of 96.58%, AUC of 98.29%, and specificity of 96.76%.

Second, when diagnosing breast cancer histological images with a magnification factor of 400×, the ANN with mixed features after dimensionality reduction for VGG-19 and ResNet-18 reached a precision of 95.54%, an accuracy of 96.4%, sensitivity of 96.72%, AUC of 97.35%, and specificity of 96.58%. While the ANN with mixed features before dimensionality reduction for VGG-19 and ResNet-18 reached a precision of 94.5%, an accuracy of 95.3%, sensitivity of 95.32%, AUC of 94.33%, and specificity of 95.87%.

**Figure 13:**
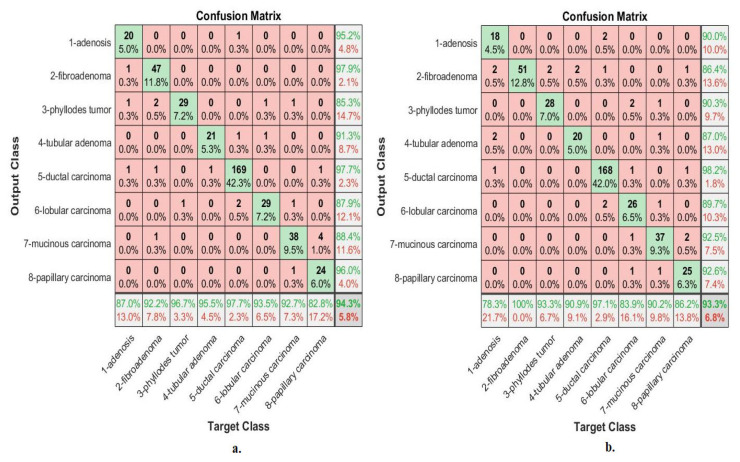
Confusion matrix for multi-classes breast cancer dataset with magnification factor 40× by ANN with selected hybrid features for VGG-19 and ResNet-18; (**a**) combined features after PCA (**b**) combined features before PCA.

Figure 13 describes the confusion matrix of performance of the ANN with selected hybrid features of the VGG-19 and ResNet-18 models of the breast cancer data set for all benign and malignant classes (multi classes) at 40× magnification factor. When ANN was fed with hybrid features of VGG-19 and ResNet-18 after reducing the dimensions, it achieves a diagnostic accuracy for all classes: for Adenosis of 87%, for Fibroadenoma of 92.2%, for Phyllodes Tumor of 96.7%, for Tubular Adenoma of 95.5%, for Ductal Carcinoma of 97.7%, for Lobular Carcinoma of 93.5%, for Mucinous Carcinoma of 92.7%, and the Papillary Carcinoma of 82.8%. While with the hybrid features of VGG-19 and ResNet-18 before reducing the dimensions, ANN achieved accuracy for all classes: for Adenosis of 78.3%, for Fibroadenoma of 100%, for Phyllodes Tumor of 93.3%, for Tubular Adenoma of 90.9%, for Ductal Carcinoma of 97.1%, for Lobular Carcinoma of 83.9%, for Mucinous Carcinoma of 90.2%, and the Papillary Carcinoma of 86.2%.

**Figure 14:**
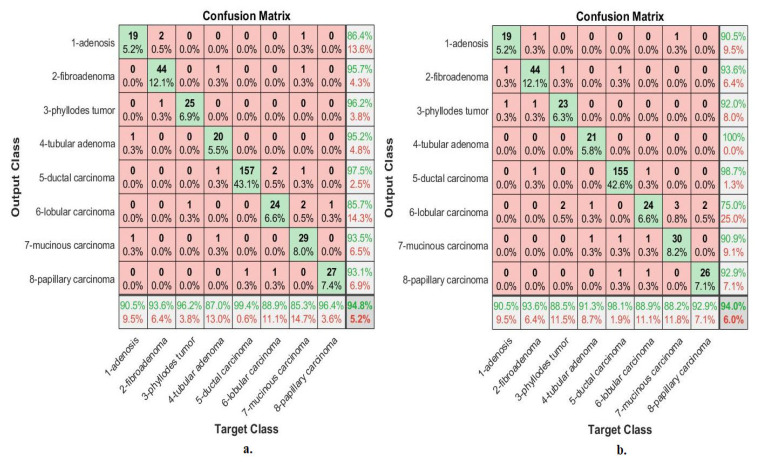
Confusion matrix for multi-classes breast cancer dataset with magnification factor 400× by ANN with selected hybrid features for VGG-19 and ResNet-18; (**a**) combined features after PCA, (**b**) combine features before PCA.

Figure 14 describes the confusion matrix of performance of the ANN with selected hybrid features of the VGG-19 and ResNet-18 models of the breast cancer data set for all benign and malignant classes (multi classes) at 400× magnification factor. When fed ANN with hybrid features of VGG-19 and ResNet-18 after reducing the dimensions, it achieves a diagnostic accuracy for all classes: for Adenosis of 90.5%, for Fibroadenoma of 93.6%, for Phyllodes Tumor of 96.2%, for Tubular Adenoma of 87%, for Ductal Carcinoma of 99.4%, for Lobular Carcinoma of 88.9%, for Mucinous Carcinoma of 85.3%, and the Papillary Carcinoma of 96.4%. While with the hybrid features of VGG-19 and ResNet-18 before reducing the dimensions, ANN achieved accuracy for all classes: for Adenosis of 90.5%, for Fibroadenoma of 93.6%, for Phyllodes Tumor of 88.5%, for Tubular Adenoma of 91.3%, for Ductal Carcinoma of 98.1%, for Lobular Carcinoma of 88.9%, for Mucinous Carcinoma of 88.2%, and the Papillary Carcinoma of 92.9%.

**Figure 15:**
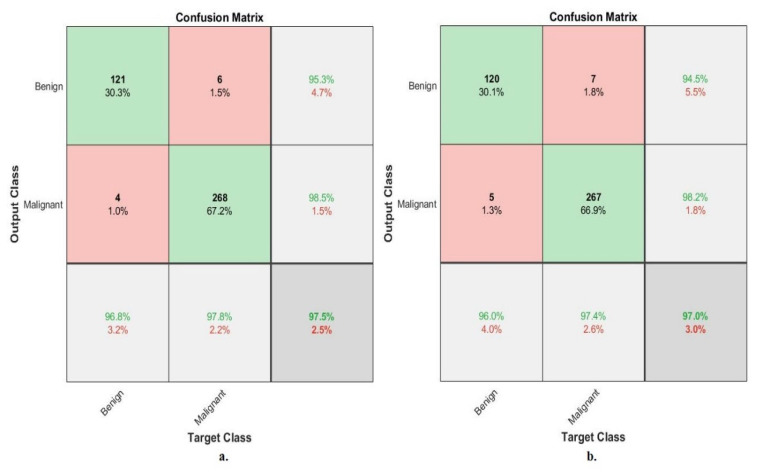
Confusion matrix for binary-classes breast cancer dataset with magnification factor 40× by ANN with selected hybrid features for VGG-19 and ResNet-18 (**a**) combine of features after PCA (**b**) combine of features before PCA.

Figure 15 describes the confusion matrix of performance of the ANN with selected hybrid features of the VGG-19 and ResNet-18 models of the breast cancer data set for benign and malignant classes (binary classes) at a 40× magnification factor. When fed ANN with hybrid features of VGG-19 and ResNet-18 after reducing the dimensions, it achieves a diagnostic accuracy for all classes: for benign of 96.8% and for malignant of 97.8%. While with the hybrid features of VGG-19 and ResNet-18 before reducing the dimensions, ANN achieved accuracy for all classes: for benign of 96% and for malignant of 97.4%.

**Figure 16:**
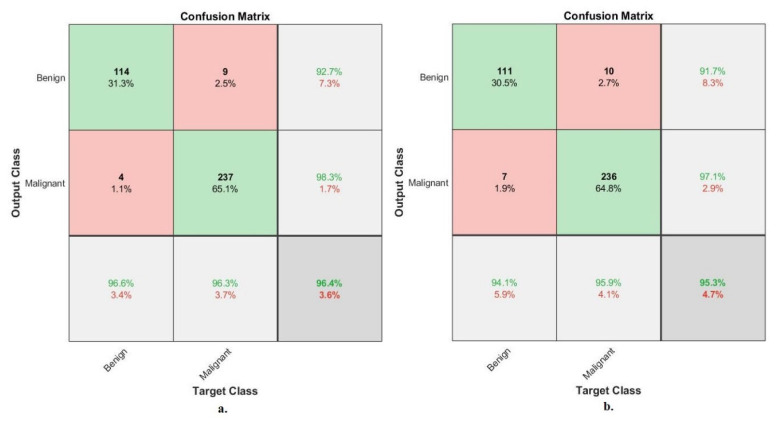
Confusion matrix for binary-classes breast cancer dataset with magnification factor 400× by ANN with selected hybrid features for VGG-19 and ResNet-18 (**a**) combine of features after PCA (**b**) combine of features before PCA.

Figure 16 describes the confusion matrix of performance of the ANN with selected hybrid features of the VGG-19 and ResNet-18 models of the breast cancer data set for benign and malignant classes (binary classes) at a 400× magnification factor. When fed ANN with hybrid features of VGG-19 and ResNet-18 after reducing the dimensions, it achieves a diagnostic accuracy for all classes: for benign of 96.6%, for malignant of 96.3%. While with the hybrid features of VGG-19 and ResNet-18 before reducing the dimensions, ANN achieved accuracy for all classes: for benign of 94.1%, for malignant of 95.9%.

### 4.6. Results of ANN According to the Features of CNN and Handcrafted

The section summarizes the results obtained by the ANN when fed with low-dimensional hybrid features from VGG-19 and handcrafted features and saved in vectors, and low-dimensional hybrid features from ResNet-18 and handcrafted features and saved in vectors, for analysis of histological images for early diagnosis and discrimination between benign and malignant breast cancer. The ANN receives and splits feature vectors to train and validate the system and test the system on new samples.

**Figure 17:**
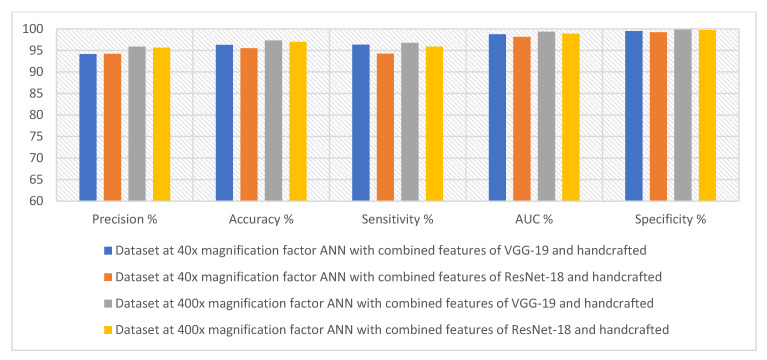
Performance of ANN with radiological features for diagnosing breast cancer and distinguishing between its benign and malignant types.

Table 7 and Figure 17 summarize the performance of the ANN with hybrid features of CNN models with handcrafted features for diagnosing breast cancer dataset for all benign and malignant classes (multi classes) at magnification factors of 40× and 400×. It should be noted that the methodology has two systems first, combining low dimensional features of VGG-19 with handcrafted features and sending them to ANN for classification. Second, combined low-dimensionality features of ResNet-18 with handcrafted features and sent them to ANN for classification.

First, when diagnosing breast cancer histological images with a magnification factor of 40×, the ANN with combined selected features of VGG-19 and handcrafted reached a precision of 94.18%, an accuracy of 96.3%, sensitivity of 96.33%, AUC of 98.74%, and specificity of 99.5%. While the ANN with combined selected features of ResNet-18 and handcrafted reached a precision of 94.22%, an accuracy of 95.5%, sensitivity of 94.25%, AUC of 98.14%, and specificity of 99.25%.

Second, when diagnosing breast cancer histological images with a magnification factor of 400×, the ANN with combined selected features of VGG-19 and handcrafted reached a precision of 95.86%, an accuracy of 97.3%, sensitivity of 96.75%, AUC of 99.37%, and specificity of 99.81%. While the ANN with combined selected features of ResNet-18 and handcrafted reached a precision of 95.63%, an accuracy of 97%, sensitivity of 95.88%, AUC of 98.87%, and specificity of 99.72%.

Table 8 summarizes the performance of the ANN with hybrid features of CNN models with handcrafted features for diagnosing breast cancer dataset for all benign and malignant classes (binary classes) at magnification factors of 40× and 400×. It should be noted that the methodology has two systems first, combining low dimensional features of VGG-19 with handcrafted features and sending them to ANN for classification. Second, combined low-dimensionality features of ResNet-18 with handcrafted features and sent them to ANN for classification.

First, when diagnosing breast cancer histological images with a magnification factor of 40×, the ANN with combined selected features of VGG-19 and handcrafted reached a precision of 99.61%, an accuracy of 99.5%, sensitivity of 98.78%, AUC of 99.46%, and specificity of 99.35%. While the ANN with combined selected features of ResNet-18 and handcrafted reached a precision of 99.37%, an accuracy of 99%, sensitivity of 98.53%, AUC of 99.14%, and specificity of 99.61%.

Second, when diagnosing breast cancer histological images with a magnification factor of 400×, the ANN with combined selected features of VGG-19 and handcrafted reached a precision of 99.74%, an accuracy of 99.7%, sensitivity of 100%, AUC of 99.85%, and specificity of 100%. While the ANN with combined selected features of ResNet-18 and handcrafted reached a precision of 99.23%, an accuracy of 99.2%, sensitivity of 99.67%, AUC of 99.68%, and specificity of 99.87%.

**Figure 18:**
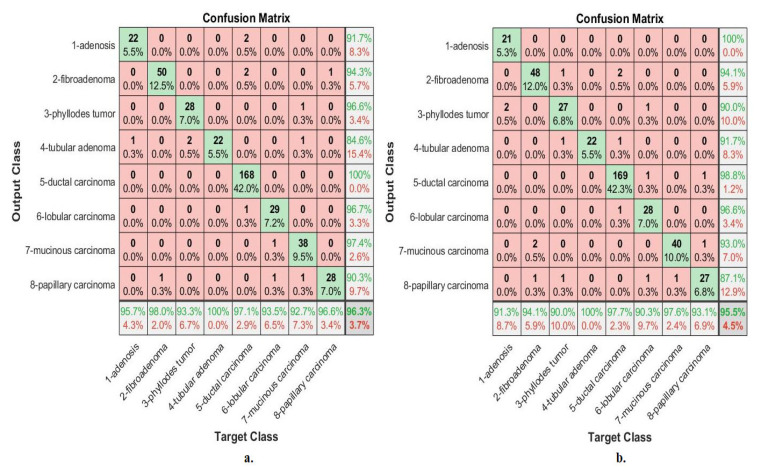
Confusion matrix for diagnosis of multi classes breast cancer dataset with magnification factor 40× using ANN with hybrid features of (**a**) VGG-19 and handcrafted feature (**b**) ResNet-18 and handcrafted feature.

Figure 18 describes the confusion matrix of performance of the ANN with selected hybrid features of the CNN (VGG-19 and ResNet-18) and handcrafted features of the breast cancer data set for all benign and malignant classes (multi classes) with 40× magnification factor. When ANN was fed with hybrid features of VGG-19 and handcrafted features, it achieves a diagnostic accuracy for all classes: for Adenosis of 95.7%, for Fibroadenoma of 98%, for Phyllodes Tumor of 93.3%, for Tubular Adenoma of 100%, for Ductal Carcinoma of 97.1%, for Lobular Carcinoma of 93.5%, for Mucinous Carcinoma of 92.7%, and the Papillary Carcinoma of 96.6%. While with the hybrid features of ResNet-18 and handcrafted features, ANN achieved accuracy for all classes: for Adenosis of 91.3%, for Fibroadenoma of 94.1%, for Phyllodes Tumor of 90%, for Tubular Adenoma of 100%, for Ductal Carcinoma of 97.7%, for Lobular Carcinoma of 90.3%, for Mucinous Carcinoma of 97.6%, and the Papillary Carcinoma of 93.1%.

**Figure 19:**
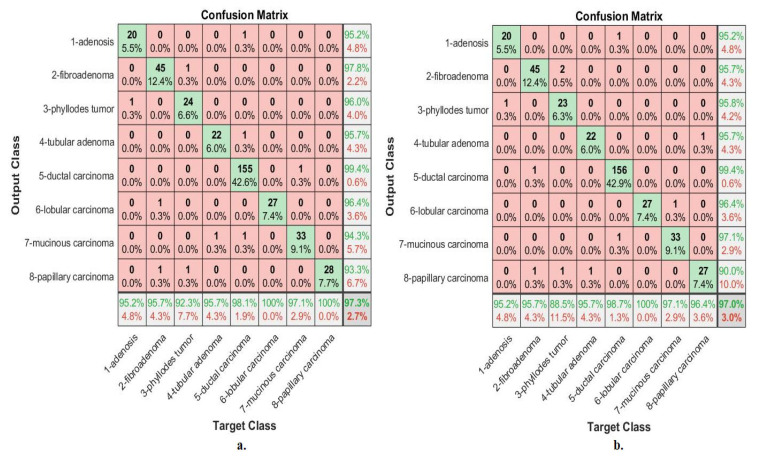
Confusion matrix for diagnosis of multi classes breast cancer dataset with magnification factor 400× using ANN with hybrid features of (**a**) VGG-19 and handcrafted feature (**b**) ResNet-18 and handcrafted feature.

Figure 19 describes the confusion matrix of performance of the ANN with selected hybrid features of the CNN (VGG-19 and ResNet-18) and handcrafted features of the breast cancer data set for all benign and malignant classes (multi classes) with 400× magnification factor. When ANN was fed with hybrid features of VGG-19 and handcrafted features, it achieves a diagnostic accuracy for all classes: for Adenosis of 95.2%, for Fibroadenoma of 95.7%, for Phyllodes Tumor of 92.3%, for Tubular Adenoma of 95.7%, for Ductal Carcinoma of 98.1%, for Lobular Carcinoma of 100%, for Mucinous Carcinoma of 97.1%, and the Papillary Carcinoma of 100%. While with the hybrid features of ResNet-18 and handcrafted features, ANN achieved accuracy for all classes: for Adenosis of 95.2%, for Fibroadenoma of 95.7%, for Phyllodes Tumor of 88.5%, for Tubular Adenoma of 95.7%, for Ductal Carcinoma of 98.7%, for Lobular Carcinoma of 100%, for Mucinous Carcinoma of 97.1%, and the Papillary Carcinoma of 96.4%.

**Figure 20:**
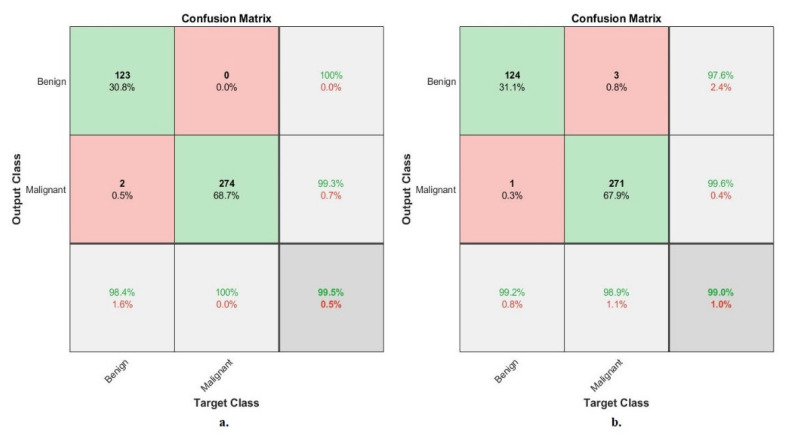
Confusion matrix for diagnosis of binary classes breast cancer dataset with magnification factor 40× using ANN with hybrid features of (**a**) VGG-19 and handcrafted feature (**b**) ResNet-18 and handcrafted feature.

Figure 20 describes the confusion matrix of performance of the ANN with selected hybrid features of the CNN (VGG-19 and ResNet-18) and handcrafted features of the breast cancer data set for benign and malignant classes (binary classes) with 40× magnification factor. When ANN was fed with hybrid features of VGG-19 and handcrafted features, it achieves a diagnostic accuracy for all classes: for benign of 98.4%, for malignant of 100%. While with the hybrid features of ResNet-18 and handcrafted features, ANN achieved accuracy for all classes: for benign of 99.2%, for malignant of 98.9%.

**Figure 21:**
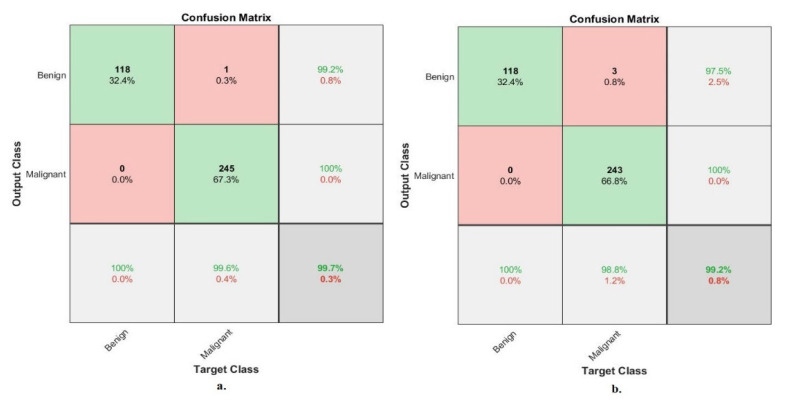
Confusion matrix for diagnosis of binary classes breast cancer dataset with magnification factor 400× using ANN with hybrid features of (**a**) VGG-19 and handcrafted feature (**b**) ResNet-18 and handcrafted feature.

Figure 21 describes the confusion matrix of performance of the ANN with selected hybrid features of the CNN (VGG-19 and ResNet-18) and handcrafted features of the breast cancer data set for benign and malignant classes (binary classes) with 400× magnification factor. When ANN was fed with hybrid features of VGG-19 and handcrafted features, it achieves a diagnostic accuracy for all classes: for benign of 100%, for malignant of 99.6%. While with the hybrid features of ResNet-18 and handcrafted features, ANN achieved accuracy for all classes: for benign of 100%, for malignant of 98.8%.

There are also some metrics to evaluate the performance of ANN for multiclass breast cancer dataset analysis with magnification factors of 40× and 400× as follows.

#### 4.6.1. Cross-Entropy

**Figure 22:**
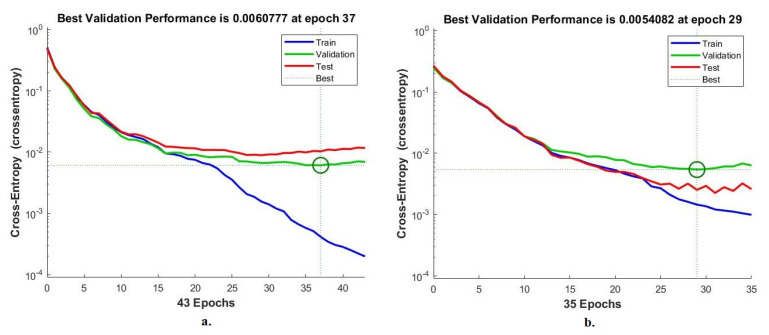
Cross-entropy for diagnosis of multi classes breast cancer dataset with magnification factor 40× using ANN with hybrid features of (**a**) VGG-19 and handcrafted feature (**b**) ResNet-18 and handcrafted feature.

Cross-entropy is an evaluative measure of ANN during the histopathological analysis of a multiclass breast cancer data set for early detection of breast cancer type. The data set goes through many epochs, and cross-entropy finds and saves the difference (error) between the actual and expected values in each epoch [48]. The network continues to work on analyzing the dataset’s images and finding the least error between the system output and the actual one. Figure 22 shows the evaluation of ANN performance for a 40× magnification multiclass breast cancer dataset through all stages through cross-entropy. The figure shows the network performance during all phases of dividing the data set in different colors. The blue color indicates the network workflow during the network training for the training data set. The green color shows the network workflow during the network validation of the validation dataset. The red color indicates the network workflow during the network test of the test data set. ANN, through cross-entropy, achieved a high-performance value of 0.0060777 at epoch 37 when fed hybrid features of VGG-19 + handcrafted features. While ANN achieved by cross-entropy a high-performance value of 0.0054082 at epoch 29 when provided with hybrid features of ResNet-18 + handcrafted features.

**Figure 23:**
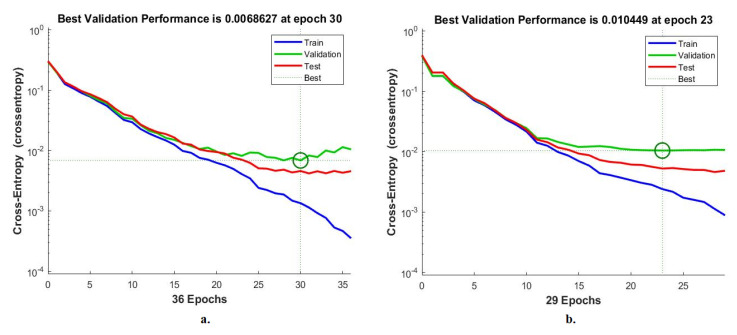
Cross-entropy for diagnosis of multi classes breast cancer dataset with magnification factor 400× using ANN with hybrid features of (**a**) VGG-19 and handcrafted feature (**b**) ResNet-18 and handcrafted feature.

Figure 23 shows the evaluation of ANN performance with a 400× magnification multiclass breast cancer dataset through all phases through cross-entropy. ANN, through cross-entropy, achieved a high-performance value of 0.0068627 at epoch 30 when fed hybrid features of VGG-19 + handcrafted features. While ANN achieved by cross-entropy a high-performance value of 0.010449 at epoch 23 when provided with hybrid features of ResNet-18 + handcrafted features.

#### 4.6.2. Gradient and Validation Checks

**Figure 24:**
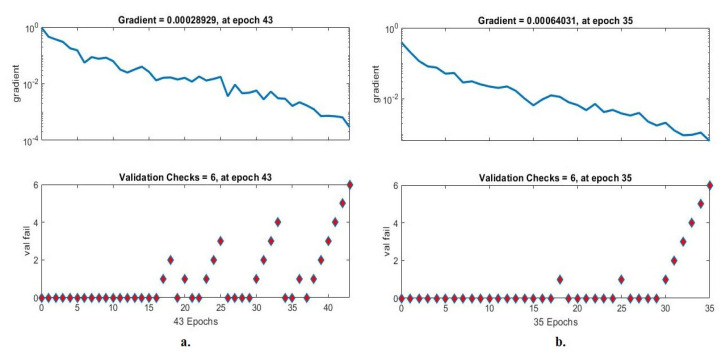
Gradient and validation for diagnosis of multi classes breast cancer dataset with magnification factor 40× using ANN with hybrid features of (**a**) VGG-19 and handcrafted feature (**b**) ResNet-18 and handcrafted feature.

Gradient and validation are an evaluative measure of ANN during histopathological image analysis of a multiclass breast cancer dataset for early detection of breast cancer type. The data set goes through many epochs, and gradient and validation detect failures in each epoch [49]. Figure 24 shows the evaluation of ANN performance for a multiclass breast cancer dataset with 40× magnification. ANN achieved through gradient the best value at epoch 43 with a value of 0.0002829 and at the same epoch validation checks with a value of 6 when fed with the radiometric features of VGG-19 + handcrafted. ANN with gradient achieved the best value at epoch 35 with a value of 0.00064031 and at the same epoch validation checks with a value of 6 when fed with the hybrid features of ResNet-18 and handcrafted.

**Figure 25:**
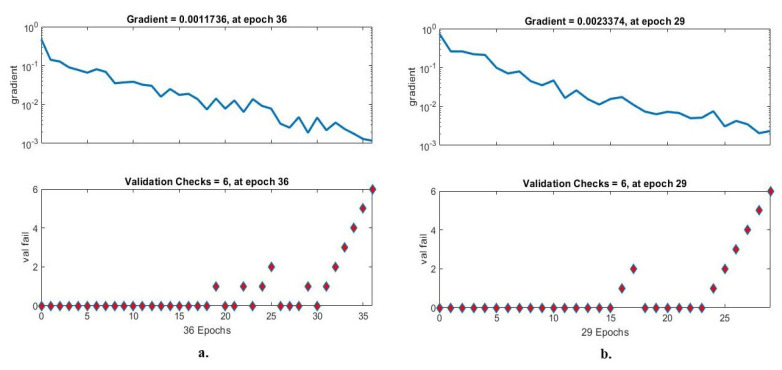
Gradient and validation for diagnosis of multi classes breast cancer dataset with magnification factor 400× using ANN with hybrid features of (**a**) VGG-19 and handcrafted feature (**b**) ResNet-18 and handcrafted feature.

Figure 25 shows the evaluation of ANN performance for a multiclass breast cancer dataset with 400× magnification. ANN achieved by gradient the best value at epoch 36 with a value of 0.0011736 and at the same epoch validation checks with a value of 6 when fed with the radiometric features of VGG-19 + handcrafted. ANN with gradient achieved the best value at epoch 29 with a value of 0.0023374 and at the same epoch validation checks with a value of 6 when fed with the hybrid features of ResNet-18 and handcrafted.

#### 4.6.3. Error Histogram

**Figure 26:**
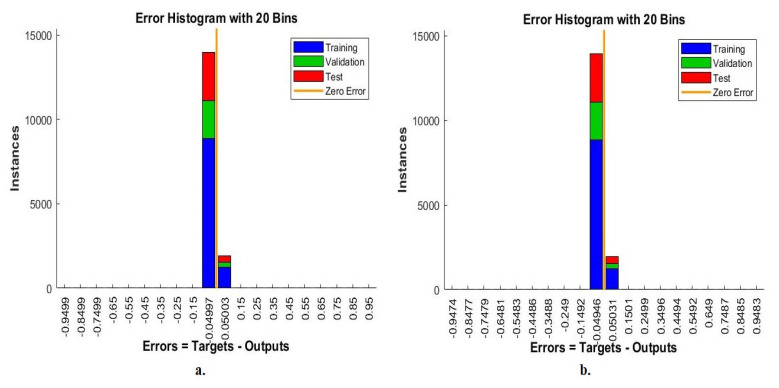
Error histogram for diagnosis of multi classes breast cancer dataset with magnification factor 40× using ANN with hybrid features of (**a**) VGG-19 and handcrafted feature (**b**) ResNet-18 and handcrafted feature.

Error histogram is an evaluative measure of ANN during the histopathological images analysis of a multiclass breast cancer dataset for early detection of breast cancer type. The data set goes through many epochs and detects the difference (error) between the actual and expected values in each epoch. The network continues to work on analyzing the dataset’s images and finding the least error between the system output and the actual image based on instances. Figure 26 shows the evaluation of ANN performance for a multiclass breast cancer dataset with 40× magnification through all stages set for data with different colors. Blue indicates the workflow of the ANN through the data set training. Green shows the workflow of the ANN through the data set validation. Red indicates ANN’s workflow through data set testing [50]. ANN, through its error histogram, achieved the best value for a network between 20 bins of −0.9499 and 0.95 when fed with the hybrid features of VGG-19 + handcrafted features. ANN, through its error histogram, achieved the best value for a network between 20 bins of −0.9474 and 0.9483 when fed with the hybrid features of ResNet-18 + handcrafted features.

**Figure 27:**
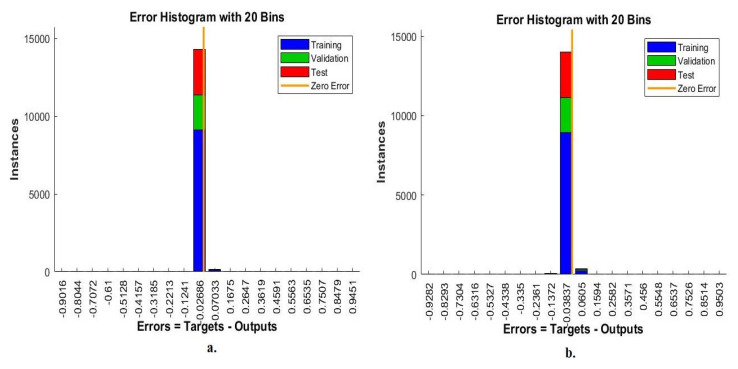
Error histogram for diagnosis of multi classes breast cancer dataset with magnification factor 400× using ANN with hybrid features of (**a**) VGG-19 and handcrafted feature (**b**) ResNet-18 and handcrafted feature.

Figure 27 shows the evaluation of ANN performance for a multiclass breast cancer dataset with 400× magnification across all stages of a dataset. ANN, through its error histogram, achieved the best value for a network between 20 bins of −0.9016 and 0.9451 when fed with the hybrid features of VGG-19 + handcrafted features. ANN, through its error histogram, achieved the best value for a network between 20 bins of −0.9282 and 0.9503 when fed with the hybrid features of ResNet-18 + handcrafted features.

## 5. Discussion the Results of the Systems and Comparison

This study presented the development of different systems with various methodologies for diagnosing histological images of breast cancer and distinguishing between types of malignant and benign tumors. There is a similarity between the characteristics of benign and malignant tumors, especially in the early stages [51]. Therefore, all methodologies focused on extracting the hidden attributes from many methods, deleting the recurring features, and saving the critical features only to reduce the dimensions and then merge them. Three methodologies were developed. Each methodology has two systems for early diagnosis of histological images of breast cancer [38] for magnification factors 40× and 400×. Each system was evaluated by images of the multi-classes and binary classes of breast cancer data set for 40× and 400× magnification factors.

Histological images of breast cancer were improved to delete artifacts and to show the edges of the affected tissue.

The first methodology consists of two systems for diagnosing and distinguishing between benign and malignant types of breast cancer for multi and binary classes of the magnification factors 40× and 400×. Features were extracted by VGG-19 and ResNet-18 separately. Redundant and unnecessary features were omitted by PCA and saved in vectors. The vectors with significant features and low-dimensional of the 40× and 400× magnification factors of the multi and binary classes of breast cancer data set were classified by ANN. First, with multi and binary classes of breast cancer data set of 40× magnification factors, ANN with the features of VGG-19 reached an accuracy of 92.3%. While with a magnification factor of 400×, with the features of the VGG-19, ANN achieved an accuracy of 91.8%.

In contrast, with a magnification factor of 40×, ANN, with the features of ResNet-18, achieved an accuracy of 92%. Whereas, with a magnification factor of 400×, ANN, with the features of ResNet-18, achieved an accuracy of 90.1%. Second, with binary classes of breast cancer dataset with a magnification factor of 40×, ANN with the features of VGG-19 reached an accuracy of 95%. While with a magnification factor of 400×, ANN, with the features of the VGG-19, achieved an accuracy of 95.1%. In contrast, with a magnification factor of 40×, ANN, with the features of ResNet-18, achieved an accuracy of 94.2%. Whereas, with a magnification factor of 400×, ANN, with the features of ResNet-18, achieved an accuracy of 94%.

The second methodology consists of two systems for diagnosing and distinguishing between benign and malignant types of breast cancer for multi and binary classes with magnification factors 40× and 400×. Features were extracted by VGG-19 and ResNet-18. Redundant and unnecessary features of high dimensionality reduction were omitted by PCA, and features of the two models before and after dimensionality reduction were combined. Vectors with hybrid features of VGG-19 and ResNet-18 before and after PCA were classified for 40× and 400× magnification factors for the multi- and binary classes of breast cancer data set by ANN. First, with a multiclass dataset with a magnification factor of 40×, with the hybrid features of VGG-19 and ResNet-18 after reducing the dimensions, ANN reached an accuracy of 94.3%. Whereas with a magnification factor of 400×, with the hybrid features of VGG-19 and ResNet-18 after reducing the dimensions, ANN reached an accuracy of 94.8%.

In contrast, with a magnification factor of 40×, ANN with the hybrid features of VGG-19 and ResNet-18 before reducing the dimensions reached an accuracy of 93.3%. While with a magnification factor of 400×, with the hybrid features of VGG-19 and ResNet-18 before reducing the dimensions, ANN reached an accuracy of 94%.

Second, with the binary classes of the breast cancer dataset with a magnification factor of 40×, with the hybrid features of VGG-19 and ResNet-18 after reducing the dimensions, ANN reached an accuracy of 97.5%. Whereas with a magnification factor of 400×, with the hybrid features of VGG-19 and ResNet-18 after reducing the dimensions, ANN reached an accuracy of 96.4%.

In contrast, with a magnification factor of 40×, ANN with the hybrid features of VGG-19 and ResNet-18 before reducing the dimensions reached an accuracy of 97%. While with a magnification factor of 400×, with the hybrid features of VGG-19 and ResNet-18 before reducing the dimensions, ANN reached an accuracy of 95.3%.

The third methodology consists of two systems for diagnosing and distinguishing benign and malignant breast cancer types of multi and binary classes with magnifications of 40× and 400×. The hybrid features were extracted by VGG-19 and ResNet-18 separately and combined with the handcrafted features. The redundant and unnecessary features of the VGG-19 and ResNet-18 models were removed before they were combined with the handcrafted features. Hybrid-featured vectors of 40× and 400× magnification factors were classified for the multi and binary classes of breast cancer dataset using ANN.

First, with a multiclass breast cancer dataset of a magnification factor of 40×, with the hybrid features of VGG-19 and handcrafted, ANN reached an accuracy of 96.3%. Whereas with a magnification factor of 400×, with the hybrid features of VGG-19 and handcrafted, ANN reached an accuracy of 97.3%.

In contrast, with a magnification factor of 40×, ANN with the hybrid features of ResNet-18 and handcrafted, reached an accuracy of 95.5%. While with a magnification factor of 400×, ANN with the hybrid features of ResNet-18 and handcrafted, reached an accuracy of 97%.

Second, with the binary classes of the breast cancer dataset with a magnification factor of 40×, ANN with the hybrid features of VGG-19 and handcrafted, reached an accuracy of 99.5%. Whereas with a magnification factor of 400×, ANN with the hybrid features of VGG-19 and handcrafted, reached an accuracy of 99.7%.

In contrast, with a magnification factor of 40×, ANN with the hybrid features of ResNet-18 and handcrafted, reached an accuracy of 99%. While with a magnification factor of 400×, ANN with the hybrid features of ResNet-18 and handcrafted, reached an accuracy of 99.2%.

**Figure 28:**
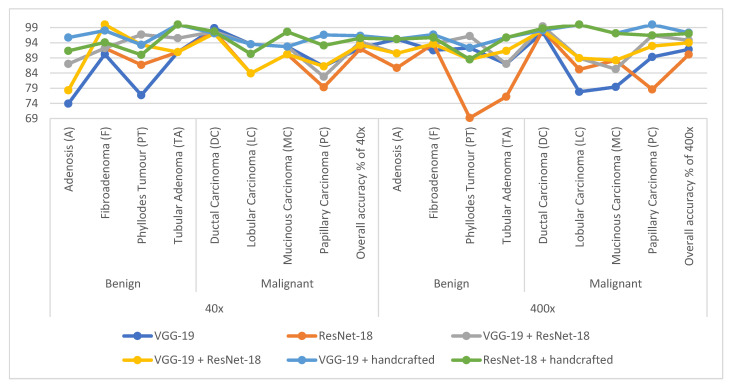
Display the performance of systems for histological diagnosis of multi-classes breast cancer with magnification factors 40× and 400×.

Table 9 and Figure 28 discuss the performance of the systems for diagnosing histological images for early detection of breast cancer and distinguishing between its malignant and benign types. The table shows the accuracy achieved by each system at the level of each type of tumor with a multi-class data set for magnification factors 40× and 400×. For the adenosis class, the best diagnosis was achieved 95.7% by ANN with VGG-19 features + handcrafted features of images at a magnification factor of 40×. For the Fibroadenoma class, the best diagnosis was achieved 100% by ANN with the hybrid features of VGG-19 + ResNet-18 for images with a magnification factor of 40×. For the Phyllodes Tumor class, the best diagnosis of 96.7% was achieved by ANN with the hybrid features of VGG-19 + ResNet-18 for images with a magnification factor of 40×. For the Tubular Adenoma class, the best diagnosis was reached by 100% by ANN with hybrid features of both systems for images with a magnification factor of 40×. For the Ductal Carcinoma class, the best diagnosis of 99.4% was achieved by ANN with the hybrid features of VGG-19 + ResNet-18 for 400× magnification factor images. For the lobular carcinoma class, the best diagnosis achieved was 100% by ANN with hybrid features of both systems for images with a magnification factor of 400×. For the Mucinous Carcinoma class, the best diagnosis achieved was at 97.6% by ANN with ResNet-18 + handcrafted features of 40× magnification factor images. For the Papillary Carcinoma class, the best diagnosis was achieved 100% by ANN with VGG-19 features + handcrafted features of images at a magnification factor of 400×.

**Figure 29:**
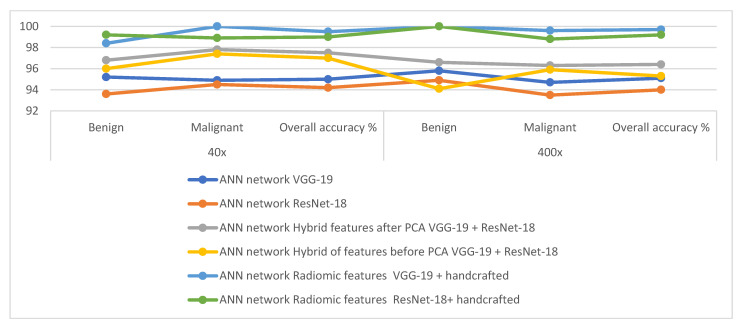
Display the performance of systems for histological diagnosis of binary classes breast cancer with magnification factors 40× and 400×.

Table 10 and Figure 29 discuss the performance of the histological images systems for the early detection of breast cancer. The table presents the accuracy achieved by each system as malignant and benign with binary classes data set for magnification factors 40× and 400×. For the benign tumors, the best diagnosis achieved was 100% by ANN with hybrid features of both systems for images with a magnification factor of 400×. For the malignant tumors, the best diagnosis achieved was 100% by ANN with features of VGG-19 + handcrafted features of images at a magnification factor of 40×.

## 6. Conclusions

The number of breast cancer cases among women, both benign and malignant, is increasing every year, posing a threat to women’s lives. In this study, several systems were developed to diagnose histological images of multi and binary classes of breast cancer datasets with magnification factors of 40× and 400×. The diversity of colors of medical fluids added to biopsies is a challenge, so the images have been improved. Techniques in this study are divided into three techniques; each has two systems for the diagnosis of multi and binary classes of breast cancer datasets for magnification factors 40× and 400×. The first technique analyzed and differentiated breast cancer dataset by ANN with features selected from VGG-19 and ResNet-18 separately. The second technique analyzed and discriminated breast cancer dataset by ANN with combined features of VGG-19 and ResNet-18 models. It should be noted that the feature collection process was done before and after deleting the redundant features and selecting the critical features to reduce the dimensions by PCA. The third technique diagnosed and discriminated the types of breast cancer dataset by ANN with hybrid features. The systems achieved satisfactory results for diagnosing multi and binary datasets for 40× and 400× magnification factors. When analyzing a multiclass data set, ANN with hybrid features of VGG-19 and handcrafted reached a precision of 95.86%, an accuracy of 97.3%, sensitivity of 96.75%, AUC of 99.37%, and specificity of 99.81% with histological images at magnification factor 400×. When analyzing a binary classes data set, ANN with hybrid features of VGG-19 and handcrafted features reached a precision of 99.74%, an accuracy of 99.7%, sensitivity of 100%, AUC of 99.85%, and specificity of 100% with histological images at a magnification factor 400×. We conclude that the best results were achieved by ANN when integrating the features of VGG-19 networks with the handcrafted features.

## Figures and Tables

**Table 1:** Distribution of histological images of the BreakHis data set according to tumor types and magnification factor.

Classes	Types	Magnification				
		40×	100×	200×	400×	Total
Benign	Adenosis (A)	114	113	111	106	444
	Fibroadenoma (F)	253	260	264	237	1014
	Phyllodes Tumor (PT)	149	150	140	130	569
	Tubular Adenoma (TA)	109	121	108	115	453
Malignant	Ductal Carcinoma (DC)	864	903	896	788	3451
	Lobular Carcinoma (LC)	156	170	163	137	626
	Mucinous Carcinoma (MC)	205	222	196	169	792
	Papillary Carcinoma (PC)	145	142	135	138	560
Total		1995	2081	2013	1820	7909

**Table 2:** Balancing of histological image data of breast cancer.

Types	Phase	Training Dataset of 40×		Training Dataset of 400×	
	Classes	Bef-Aug	Aft-Aug	Bef-Aug	Aft-Aug
Benign	Adenosis (A)	73	1606	68	1496
	Fibroadenoma (F)	162	1620	152	1520
	Phyllodes Tumor (PT)	95	1615	83	1494
	Tubular Adenoma (TA)	70	1610	74	1480
Malignant	Ductal Carcinoma (DC)	553	1659	504	1512
	Lobular Carcinoma (LC)	100	1600	88	1496
	Mucinous Carcinoma (MC)	131	1572	108	1404
	Papillary Carcinoma (PC)	93	1581	88	1496

**Table 3:** Results of ANN performance with selected features of VGG-19 and ResNet-18 from multi-classes dataset.

Dataset	Dataset at 40× Magnification Factor		Dataset at 400× Magnification Factor	
Measure	ANN with VGG-19 Features	ANN with ResNet-18 Features	ANN with VGG-19 Features	ANN with ResNet-18 Features
Precision %	89.25	88.38	88.13	86.63
Accuracy %	92.3	92	91.8	90.1
Sensitivity %	88.12	88.45	88.62	84.38
AUC %	93.15	91.48	94.25	91.21
Specificity %	98.88	98.75	97.95	98.63

**Table 4:** Results of ANN performance with selected features of VGG-19 and ResNet-18 with binary classes dataset.

Dataset	Dataset at 40× Magnification Factor		Dataset at 400× Magnification Factor	
Measure	ANN with VGG-19 Features	ANN with ResNet-18 Features	ANN with VGG-19 Features	ANN with ResNet-18 Features
Precision %	93.5	93.28	94.31	92.5
Accuracy %	95	94.2	95.1	94
Sensitivity %	95.17	94.66	95.72	94.43
AUC %	96.34	95.87	96.78	93.82
Specificity %	95.58	95.41	96.35	95.73

**Table 5:** Results of ANN performance with mixed features of CNN with multi classes dataset.

Dataset	Dataset at 40× Magnification Factor		Dataset at 400× Magnification Factor	
Measure	ANN with Mixed Features of CNN after PCA	ANN with Mixed Features of CNN before PCA	ANN with Mixed Features of CNN after PCA	ANN with Mixed Features of CNN before PCA
Precision %	92.38	90.88	93.23	91.75
Accuracy %	94.3	93.3	94.8	94
Sensitivity %	92.29	89.86	92.41	91.38
AUC %	96.88	93.79	96.17	95.89
Specificity %	99.25	98.86	99.13	99.1

**Table 6:** Results of ANN performance with mixed features of CNN with binary classes dataset.

Dataset	Dataset at 40× Magnification Factor		Dataset at 400× Magnification Factor	
Measure	ANN with Mixed Features of CNN after PCA	ANN with Mixed Features of CNN before PCA	ANN with Mixed Features of CNN after PCA	ANN with Mixed Features of CNN before PCA
Precision %	97.34	96.12	95.54	94.5
Accuracy %	97.5	97	96.4	95.3
Sensitivity %	97.82	96.58	96.72	95.32
AUC %	98.86	98.29	97.35	94.33
Specificity %	97.91	96.76	96.58	95.87

**Table 7:** Results of ANN performance with combined features of CNN with handcrafted features of multi classes dataset.

Dataset	Dataset at 40× Magnification Factor		Dataset at 400× Magnification Factor	
Measure	ANN with Combined Features of VGG-19 and Handcrafted	ANN with Combined Features of ResNet-18 and Handcrafted	ANN with Combined Features of VGG-19 and Handcrafted	ANN with Combined Features of ResNet-18 and Handcrafted
Precision %	94.18	94.22	95.86	95.63
Accuracy %	96.3	95.5	97.3	97
Sensitivity %	96.33	94.25	96.75	95.88
AUC %	98.74	98.14	99.37	98.87
Specificity %	99.5	99.25	99.81	99.72

**Table 8:** Results of ANN Performance with combined features of CNN with handcrafted features of binary classes dataset.

Dataset	Dataset at 40× Magnification Factor		Dataset at 400× Magnification Factor	
Measure	ANN with Combined Features of VGG-19 and Handcrafted	ANN with Combined Features of ResNet-18 and Handcrafted	ANN with Combined Features of VGG-19 and Handcrafted	ANN with Combined Features of ResNet-18 and Handcrafted
Precision %	99.61	99.37	99.74	99.23
Accuracy %	99.5	99	99.7	99.2
Sensitivity %	98.78	98.53	100	99.67
AUC %	99.46	99.14	99.85	99.68
Specificity %	99.35	99.61	100	99.87

**Table 9:** Summary of results of systems for diagnosing histological images of multi-classes breast cancer with magnification factors 40× and 400×.

Magnification Factor	Classes	Techniques	ANN Network					
		Features	VGG- 19	ResNet- 18	Hybrid Features after PCA	Hybrid of Features before PCA	Hybrid Features	
		Types			VGG-19 + ResNet-18	VGG-19 + ResNet-18	VGG-19 + Handcrafted	ResNet-18 + Handcrafted
40×	Benign	Adenosis (A)	73.9	87	87	78.3	95.7	91.3
		Fibroadenoma (F)	90.2	92.2	92.2	100	98	94.1
		Phyllodes Tumor (PT)	76.7	86.7	96.7	93.3	93.3	90
		Tubular Adenoma (TA)	90.9	90.9	95.5	90.9	100	100
	Malignant	Ductal Carcinoma (DC)	98.8	97.7	97.7	97.1	97.1	97.7
		Lobular Carcinoma (LC)	93.5	83.9	93.5	83.9	93.5	90.3
		Mucinous Carcinoma (MC)	92.7	90.2	92.7	90.2	92.7	97.6
		Papillary Carcinoma (PC)	86.2	79.3	82.8	86.2	96.6	93.1
Overall accuracy %			92.3	92	94.3	93.3	96.3	95.5
400×	Benign	Adenosis (A)	95.2	85.7	90.5	90.5	95.2	95.2
		Fibroadenoma (F)	91.5	93.6	93.6	93.6	96.7	95.7
		Phyllodes Tumor (PT)	92.3	69.2	96.2	88.5	92.3	88.5
		Tubular Adenoma (TA)	87	76.2	87	91.3	95.7	95.7
	Malignant	Ductal Carcinoma (DC)	97.5	98.1	99.4	98.1	98.1	98.7
		Lobular Carcinoma (LC)	77.8	85.2	88.9	88.9	100	100
		Mucinous Carcinoma (MC)	79.4	88.2	85.3	88.2	97.1	97.1
		Papillary Carcinoma (PC)	89.3	78.6	96.4	92.9	100	96.4
Overall accuracy %			91.8	90.1	94.8	94	97.3	97

**Table 10:** Summary of results of systems for diagnosing histological images of binary classes breast cancer with magnification factors 40× and 400×.

Techniques	Magnification Factor		40×			400×		
	Features		Benign	Malignant	Overall Accuracy %	Benign	Malignant	Overall Accuracy %
ANN network	VGG-19		95.2	94.9	95	95.8	94.7	95.1
	ResNet-18		93.6	94.5	94.2	94.9	93.5	94
	Hybrid features after PCA	VGG-19 + ResNet-18	96.8	97.8	97.5	96.6	96.3	96.4
	Hybrid of features before PCA	VGG-19 + ResNet-18	96	97.4	97	94.1	95.9	95.3
	Hybrid features	VGG-19 + handcrafted	98.4	100	99.5	100	99.6	99.7
		ResNet-18+ handcrafted	99.2	98.9	99	100	98.8	99.2

## Data Availability

The histological images supporting the performance of the system results were obtained from the publicly available BreakHis breast cancer dataset at: https://www.kaggle.com/ambarish/breakhis.(accessed on 19 December 2022).
